# A mixed-effects stochastic model reveals clonal dominance in gene therapy safety studies

**DOI:** 10.1186/s12859-023-05269-1

**Published:** 2023-06-02

**Authors:** Luca Del Core, Danilo Pellin, Ernst C. Wit, Marco A. Grzegorczyk

**Affiliations:** 1 Bernoulli Institute for Mathematics, Computer Science and Artificial Intelligence, University of Groningen, Groningen, The Netherlands; 2 School of Mathematical Sciences, University of Nottingham, Nottingham, UK; 3 Harvard Medical School, Harvard University, Boston, MA USA; 4 Institute of Computing, Università della Svizzera italiana, Lugano, Switzerland

**Keywords:** Stochastic reaction networks, Mixed-effects models, E-M algorithm, $\tau$-Leaping, Gene therapy, Clonal dominance

## Abstract

**Background:**

Mathematical models of haematopoiesis can provide insights on abnormal cell expansions (clonal dominance), and in turn can guide safety monitoring in gene therapy clinical applications. Clonal tracking is a recent high-throughput technology that can be used to quantify cells arising from a single haematopoietic stem cell ancestor after a gene therapy treatment. Thus, clonal tracking data can be used to calibrate the stochastic differential equations describing clonal population dynamics and hierarchical relationships in vivo.

**Results:**

In this work we propose a random-effects stochastic framework that allows to investigate the presence of events of clonal dominance from high-dimensional clonal tracking data. Our framework is based on the combination between stochastic reaction networks and mixed-effects generalized linear models. Starting from the Kramers–Moyal approximated Master equation, the dynamics of cells duplication, death and differentiation at clonal level, can be described by a local linear approximation. The parameters of this formulation, which are inferred using a maximum likelihood approach, are assumed to be shared across the clones and are not sufficient to describe situation in which clones exhibit heterogeneity in their fitness that can lead to clonal dominance. In order to overcome this limitation, we extend the base model by introducing random-effects for the clonal parameters. This extended formulation is calibrated to the clonal data using a tailor-made expectation-maximization algorithm. We also provide the companion 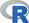 package RestoreNet, publicly available for download at https://cran.r-project.org/package=RestoreNet.

**Conclusions:**

Simulation studies show that our proposed method outperforms the state-of-the-art. The application of our method in two in-vivo studies unveils the dynamics of clonal dominance. Our tool can provide statistical support to biologists in gene therapy safety analyses.

**Supplementary Information:**

The online version contains supplementary material available at 10.1186/s12859-023-05269-1.

## Background

In gene therapy the correction of the defective gene(s) underlying the disease is, in principle, sufficient for inducing disease remission or even full recovery [1]. Since the blood system possesses a hierarchical structure with haematopoietic stem cells (HSCs) at its root [2], correction of large numbers of HSCs might be sufficient to eradicate a genetic disease [3, 4]. But genetic modification of large numbers of cells is associated with the higher probability of unintentional vector insertions near proto oncogenes, that may lead to insertional mutagenesis [5–7]. Insertional mutagenesis causes a significant change in clone fitness that can lead to the clones’ abnormal expansion and to an unbalanced contribution of different clones to blood cells production. Clonal dominance, characterised by the outgrowth of a small subset of clones (oligoclonality) or one clone (monoclonality) in the most extreme cases, poses serious concerns in the context of gene therapy clinical trials because they might represent the initial stage of a leukemic transformation and are in general considered negative predictors of long term therapeutic benefit.

Clonal dominance in malignant haematopoiesis has been previously identified as a consequence of a clonal competition that is corrupted by disease progression [8, 9]. However, clonal dominance has also been observed in normal haematopoiesis, even in the case of truly neutral clonal markers [10–12]. Indeed, on the basis of various mathematical models, progression of monoclonality has been discussed also for normal (non-leukaemic) stem cell systems [13–17]. While there is strong evidence for clonal selection inducing monoclonal systems in the crypts of the small intestine [18–21], such a process has not been demonstrated for the haematopoietic system yet. There are several high-throughput systems that allow to quantitatively investigate those mechanisms. In gene therapy applications, clonal tracking is performed by using permanent molecular identifier integrated in the host cell genome. In pre-clinical animal studies, these are short fragments of random or semi-random DNA stretches called barcodes, whereas in clinical setting vector integration sites are in general used. After transplantation, all the progeny deriving through cell differentiation inherits the original labels, thus allowing computational modelling to unveil population dynamics and hierarchical relationships in vivo [22–25].

Here we extend the work by [26, 27] and propose a random-effects cell differentiation network to detect the dynamics of clonal expansion from high dimensional clonal tracking data. In particular, starting from the definition of the Master equation [28], a set of Ito-type stochastic differential equations is derived to describe the first two-order moments of the process. We estimate the parameters of the Ito system from its Euler-Maruyama local linear approximation (LLA) [29] using a maximum likelihood approach. Although the base LLA model formulation has been shown to be effective in modelling cell differentiation [27], it has some limitations as it considers all clone trajectories to be iid realizations of the same underlying stochastic process, and does not take into account possible heterogeneous behaviour across the clones. Therefore, the base LLA formulation cannot be used to model clonal dominance. In this work we further increase the flexibility of the base LLA model to take into account for potential heterogeneity in clones’ behaviour in both duplication and differentiation rates. To this end we introduce random-effects for the clones inside the LLA formulation, providing a mixed-effects LLA model. Then, we use the inferred mixed-effects model to identify which clones are mainly expanding and in which cell compartments. Parameter inference in the mixed-effects formulation is performed by means of an expectation-maximization algorithm, for which we developed an efficient implementation in the 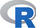 package RestoreNet. Our random-effects LLA formulation describes a stochastic process of clonal dominance on a network of cell lineages. We tested and validated our method in simulation studies, including a direct comparison with the state-of-the-art method GLS [27]. Subsequently, our method is applied to investigating the dynamics of clonal expansion in a in-vivo model of rhesus macaque haematopoiesis [23]. Finally, by analysing an in-vivo model of tumor prone mice, our method identifies the expected impact of vector genotoxicity on clonal dynamics [30].

## Methods

**Fig. 1:**
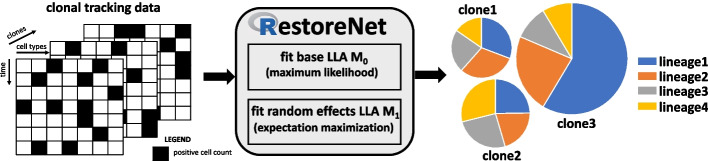
Schematic representation of the analysis: A three-dimensional clonal tracking dataset (left) is received as input from our proposed stochastic framework RestoreNet (middle). It mainly consists in two parts, such as a maximum likelihood step to infer the base LLA model, and an expectation-maximization step to infer the random-effects LLA formulation. Finally, a clonal piechart is returned, where each clone is identified by a pie whose slices are lineage specific and proportional to their expansion rates (right)

An outline of our proposed stochastic framework is as follows. RestoreNet takes a clonal tracking dataset as input, along with a set of reactions coding for cellular duplication, death and differentiation. The system of stochastic differential equations describing the clonal dynamics are translated into a generalized linear model formulation, that possibly includes clone-specific random-effects on the dynamics parameters. Subsequently, the parameters are inferred and, if an event of clonal dominance is detected, a pie-chart shows the clones that are expanding and in which cell lineage. A graphical representation of the framework is provided in Fig. 1. This section contains a concise description of the stochastic formulation of clonal dominance and the corresponding inference method. A more detailed description of the stochastic model can be found in the Additional file 1.

### A stochastic model for cell differentiation

Consistently with the definition of a stochastic quasi-reaction network of Section 1.1 of the Additional file 1, we consider a Markov process

1 $$\begin{aligned} \pmb {x}_t = (x_{1t},\dots ,x_{nt})\, , \end{aligned}$$

for a single clone and *n* cell types ($i = 1,\dots , n$) that evolve, in a time interval $(t, t + \Delta t)$, according to a set of *K* distinct biochemical reactions whose net-effect vectors $\{\pmb {v}_k\}_{k=1}^{K}$ and hazard functions $\{h_{k}(\pmb {x}_t, \pmb {\theta })\}_{k=1}^{K}$ are defined as

2 $$\begin{aligned} \pmb {v}_{k} = {\left\{ \begin{array}{ll} (\cdots \underset{i(k)}{1}\ \cdots )' \\ (\cdots -\underset{i(k)}{1}\cdots )' \\ (\cdots -\underset{i(k)}{1}\cdots \underset{j(k)}{2} \cdots )' \end{array}\right. } \quad h_{k}(\pmb {x}_t, \pmb {\theta }) = {\left\{ \begin{array}{ll} x_{i(k)t}\alpha_{i(k)} &{} \text {duplication} \\ x^2_{i(k)t}\delta_{i(k)} &{} \text {death} \\ x_{i(k)t}\lambda_{i(k)j(k)} &{} \text {differentiation} \end{array}\right. } \end{aligned}$$

where *i*(*k*) and *j*(*k*) are the cell types possibly involved in the *k*-th reaction, and 

3 $$\begin{aligned}j(k)\in \mathcal {O}(i(k))=\{j \vert \lambda _{i(k)j} > 0 \} \end{aligned} \, ,$$

where $\mathcal{O}(i)$ is called the offspring set of cell type *i*. The definitions of the hazard functions and the net-effects follow from the law of mass action, consistently with Eq. (7) of the Additional file 1. The hazard functions include a linear growth term $x_{i(k)t}\alpha_{i(k)}$ for cell lineage *i*(*k*) with a duplication rate parameter $\alpha_{i(k)} > 0$, a quadratic term $x^2_{i(k)t}\delta_{i(k)}$ for cell death of lineage *i*(*k*) with a death rate parameter $\delta_{i(k)} > 0$, and a linear term $x_{i(k)t}\lambda_{i(k)j(k)}$ describing cell differentiation from cell lineage *i*(*k*) to cell lineage $j(k) \in \mathcal {O}(i(k))$ with a differentiation rate $\lambda _{i(k)j(k)} > 0$. The vector parameter

4 $$\begin{aligned} \begin{aligned} \pmb {\theta } = \left( \alpha _1, \dots , \alpha _n, \delta _1, \dots , \delta _n, \pmb {\lambda }'_{1\mathcal {O}(1)}, \dots , \pmb {\lambda }'_{n\mathcal {O}(n)} \right) ', \end{aligned} \end{aligned}$$

appearing in the hazard functions, includes all the dynamic parameters, where $\pmb {\lambda }_{i\mathcal {O}(i)}$ is the vector of all the differentiation rates from cell lineage *i* to its offspring set $\mathcal {O}(i)$. Finally, we define the net-effect matrix and the hazard vector as

5 $$\begin{aligned} \pmb {V}&= \begin{bmatrix} \pmb {v}_{1} \cdots \pmb{v}_{K} \end{bmatrix} \in \mathbb {Z}^{n \times K}\, , \\ \pmb {h}(\pmb {x}_t, \pmb {\theta })&= \left( h_{1}(\pmb {x}_t, \pmb {\theta }), \dots , h_{K}(\pmb {x}_t, \pmb {\theta }) \right) ' \, .\end{aligned}$$

### LLA formulation of clonal dominance

Let $\pmb {y}_t = (y_{1t}, \dots ,y_{nt})'$ be the vector of the measurements collected at time *t* for a *n*-dimensional counting process $\pmb {x}_t = (x_{1t},\dots ,x_{nt})'$ obeying to a network of stochastic biochemical reactions defined by a net-effect matrix $\pmb {V} \in \mathbb {Z}^{n \times K}$, a vector parameter $\pmb {\theta } \in \mathbb {R}^{K}$ and an hazard vector $h(\pmb {x},\pmb {\theta }) = (h_1(\pmb {x},\pmb {\theta }),\dots , h_K(\pmb {x},\pmb {\theta }))'$ and let

6 $$\begin{aligned} \underbrace{\left[ \begin{array}{l} \Delta \pmb {y}_{t_0} \\ \vdots \\ \Delta \pmb {y}_{t_{T-1}} \end{array} \right] }_{\Delta \pmb {y}}&= \underbrace{\left[ \begin{array}{l} \pmb {M}_{t_0} \\ \vdots \\ \pmb {M}_{t_{T-1}} \end{array} \right] }_{\pmb {M}} \pmb {\theta } + \pmb {\varepsilon } \, , \quad \pmb {\varepsilon } \sim \mathcal {N}_{nT} \left( \pmb {0}, \overbrace{\underbrace{\left[ \begin{array}{lll} \pmb {W}_{t_{0}}(\pmb {\theta }) &{} &{} \\ &{} \ddots &{} \\ &{} &{} W_{t_{T-1}}(\pmb {\theta }) \end{array} \right] }_{\pmb {W}(\pmb {\theta })} + \sigma ^{2}\pmb {I}_{nT}}^{\pmb {\Sigma }(\pmb {\theta },\sigma ^{2})} \right) \, , \end{aligned}$$

be the local linear approximation of the Kramers-Moyal approximated Master equation (see Section 1.3 of the Additional file 1 for details) where

7 $$\begin{aligned}&\Delta \pmb {y}_t = \overbrace{\pmb {V}\left[ {\begin{matrix} \prod _{i=1}^{n} {{y_{it}}\atopwithdelims (){r_{1i}}} &{} &{} \\ &{} \ddots &{} \\ {} &{} &{} \prod _{i=1}^{n} {{y_{it}}\atopwithdelims (){r_{Ki}}} \end{matrix}} \right] \Delta t}^{\pmb {M}_t} \underbrace{\left[ {\begin{matrix} \theta _1 \\ \vdots \\ \theta _K \end{matrix}} \right] }_{\pmb {\theta }} + \left( \underbrace{\pmb {V}\left[ {\begin{matrix} h_1(\pmb {y}_t,\pmb {\theta }) &{} &{} \\ &{} \ddots &{} \\ {} &{} &{} h_1(\pmb {y}_t,\pmb {\theta }) \end{matrix}} \right] \pmb {V}'\Delta t}_{\pmb {W}_t(\theta )} + \sigma ^2\pmb {I}_n \right) ^{1/2}\Delta \pmb {\varepsilon }_t\, , \\&\quad \Delta \pmb {\varepsilon }_t \sim \mathcal {N}_n(\pmb {0}, \pmb {I}_n)\, , \end{aligned}$$

with $\sigma ^2$ being the measurement noise variance, $\pmb {M}_t \pmb {\theta }$ the mean drift, $\pmb {W}_t(\pmb {\theta })$ the diffusion matrix, and $\Delta \pmb {y}_t = \pmb {y}_{t + \Delta t} - \pmb {y}_t$ is a finite-time increment of $\pmb {y}$ in the time interval $\Delta t$. From Eq. (6) it can be seen that all clones share the same vector parameter $\pmb {\theta }$. To infer the parameters of Eqs. (6)–(7) we developed a maximum likelihood algorithm which is fully described in Section 1.4 of the Additional file 1.

In some cases it may happen that the clones being analysed are drawn from a hierarchy of *J* different populations that possibly behave differently in terms of dynamics. In this case it might be of interest to quantify the population-average $\pmb {\theta }$ and the clonal-specific effects *u* around the average $\pmb {\theta }$ for the description of clone-specific dynamics. For achieving this goal, we extend the LLA formulation of Eq. (6) with a mixed-effects model [31] by introducing random-effects $\pmb {u}$ for the *J* distinct clones on the vector parameter $\pmb {\theta }$, leading to a random-effects stochastic reaction network (RestoreNet). The extended random-effects formulation becomes

8 $$\begin{aligned}&\Delta \pmb {y} = \underbrace{\begin{bmatrix} \pmb {M}_1 &{} &{} {\textbf {0}} \\ &{} \ddots &{} \\ {\textbf {0}} &{} &{} \pmb {M}_J \end{bmatrix}}_{\pmb {\mathbb {M}} \in \mathbb {R}^{nT \times Jp}}\pmb {u} + \pmb {\varepsilon }\, , \quad \pmb {u} \sim \mathcal {N}_{Jp} \left( \underbrace{\textbf{1}_J \otimes \pmb {\theta }}_{\pmb {\theta }_u}, \pmb {I}_J\otimes \underbrace{\begin{bmatrix} \tau _1^2 &{} &{} {\textbf {0}} \\ &{} \ddots &{} \\ {\textbf {0}} &{} &{} \tau _p^2 \end{bmatrix}}_{\pmb {\Delta }_u} \right) \, , \\&\quad \pmb {\varepsilon } \sim \mathcal {N}_{nT}(\pmb {0}, \pmb {\Sigma }(\pmb {\theta }, \sigma ^2)) \, , \end{aligned}$$

where $\pmb {\mathbb {M}}$ is the block-diagonal design matrix for the random-effects $\pmb {u}$ centered in $\pmb {\theta }$, each block $\pmb {M}_j$ is clone-specific, and $\otimes$ is the Kronecker product. As in the case of the null model of Eq. (6), we estimate $\sigma ^2$ based on data. From Section 1.5 of the Additional file 1 the conditional distribution of the random-effects $\pmb {u}$ given the data $\Delta \pmb {y}$ is

9 $$\begin{aligned} \pmb {u}\vert \Delta \pmb {y} \sim \mathcal {N}_{Jp}(E_{\pmb {u}\vert \Delta \pmb {y}; \pmb {\psi }}[\pmb {u}], V_{\pmb {u}\vert \Delta \pmb {y}; \pmb {\psi }}(\pmb {u}))\, , \end{aligned}$$

where

10 $$\begin{aligned} \begin{aligned} E_{\pmb {u}\vert \Delta \pmb {y}; \pmb {\psi }}[\pmb {u}]&= V_{\pmb {u}\vert \Delta \pmb {y}; \pmb {\psi }}(\pmb {u}) \left( \pmb {\mathbb {M}}'\pmb {\Sigma }^{-1}(\pmb {\theta },\sigma ^2)\Delta \pmb {y} + \pmb {\Delta }_u^{-1}\pmb {\theta }_u \right) \, , \\ V_{\pmb {u}\vert \Delta \pmb {y}; \pmb {\psi }}(\pmb {u})&= \left( \pmb {\mathbb {M}}' \pmb {\Sigma }^{-1}(\pmb {\theta },\sigma ^2)\pmb {\mathbb {M}} + \pmb {\Delta }_u^{-1}\right) ^{-1}\, , \end{aligned} \end{aligned}$$

and $\pmb {\psi } = (\pmb {\theta }', \sigma ^2,\tau _1^2, \dots , \tau _p^2)'$ is the vector of all the unknown parameters. Once the parameters are estimated (see next section for inference details), the conditional expectations $E_{\pmb {u}\vert \Delta \pmb {y}; \pmb {\psi }}[\pmb {u}]$ can then be used as a proxy for the clone-specific dynamic parameters. This method allows to infer clone-specific dynamics by extremely reducing the problem dimensionality from $J \cdot p$ to $2 \cdot p + 1$ ($J \gg 2$).

### Inference procedure

In order to infer the maximum likelihood estimator $\hat{\pmb {\psi }}$ for $\pmb {\psi } = (\pmb {\theta }', \sigma ^2,\tau _1^2, \dots , \tau _p^2)'$, we have developed an efficient expectation-maximization (E-M) algorithm where the collected cell increments $\Delta \pmb {y}$ and the random-effects $\pmb {u}$ take the roles of the observed and latent states respectively. The full analytical expression of the E-step function $Q(\pmb {\psi } \vert \pmb {\psi }^*) = E_{\pmb {u}\vert \Delta \pmb {y}; \pmb {\psi }^*}[\ell (\Delta \pmb {y}, \pmb {u}; \pmb {\psi } )]$ and its partial derivatives $\frac{\partial }{\partial \psi _j}Q(\pmb {\psi } \vert \pmb {\psi }^*)$ are available (see Section 1.5 of the Additional file 1). In the E-M algorithm we iteratively update the E-function $Q(\pmb {\psi } \vert \pmb {\psi }^*)$ using the current estimate $\pmb {\psi }^*$ of $\pmb {\psi }$ and then we minimize the $-Q(\pmb {\psi } \vert \pmb {\psi }^*)$ w.r.t. $\pmb {\psi }$. As the E-step function $Q(\pmb {\psi } \vert \pmb {\psi }^*)$ is non-linear and the parameters are box-constrained, we used the L-BFGS-B algorithm from the optim() base R function for optimization, to which we provided the objective function, along with its gradient $\nabla _{\pmb {\psi }} Q(\pmb {\psi } \vert \pmb {\psi }^*)$, as input. The E-M algorithm is iterated until a convergence criterion is met, that is when the relative errors of the E-step function $Q(\pmb {\psi } \vert \pmb {\psi }^*)$ and the parameters $\pmb {\psi }^*$ are lower than a predefined tolerance.

Once we get the E-M estimate $\hat{\pmb {\psi }}$ for the parameters we evaluate the goodness-of-fit of the mixed-effects model according to the conditional Akaike information criterion [32]. As every E-M algorithm, the choice of the starting point $\pmb {\psi }_s$ is very important from a computational point of view. We chose $\pmb {\psi }_s = (\pmb {\theta }'_s, \sigma ^2_s, \tau _1^2 = 0, \dots , \tau _p^2 = 0)'$ as a starting point where $(\pmb {\theta }'_s, \sigma ^2_s)$ is the optimum found in the fixed-effects LLA formulation of Eq. (6). This is a reasonable choice since we want to quantify how the dynamics $E_{\pmb {u}\vert \Delta \pmb {y}; \hat{\pmb {\psi }}}[\pmb {u}]_j$ of each clone *j* departs from the average dynamics $\pmb {\theta }_s$. With the help of simulation studies (see Results section), we empirically proved that this choice always led to a conditional expectation $E_{\pmb {u}\vert \Delta \pmb {y}; \hat{\pmb {\psi }}}[\pmb {u}]$ consistent with the true clone-specific dynamic parameters $\pmb {\theta }$. Computational details can be found in Section 1.5 of the Additional file 1. The pseudocode of the E-M algorithm is provided in Algorithm 3 of the Additional file 1. The maximum likelihood inference for the basal model and the expectation-maximization algorithm for the random-effects model are implemented in the 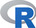 package RestoreNet, available for download at https://cran.r-project.org/package=RestoreNet.

### Model selection

The fixed-effects model $\mathcal {M}_0$ is scored according to the corrected Akaike information criterion (AIC) [33] defined as

11 $$\begin{aligned} AIC(\mathcal {M}_0) = - 2\ell _{\mathcal {M}_0}(\pmb {\theta }, \sigma ^2 \vert \Delta \pmb {y}) + \frac{2dp_{\mathcal {M}_0}}{d - p_{\mathcal {M}_0} - 1}\, , \end{aligned}$$

where $\ell _{\mathcal {M}_0}$ is the log-likelihood of the null model $\mathcal {M}_0$, $d = nT$ is the size of $\Delta \pmb {y}$, and $p_{\mathcal {M}_0}$ the corresponding number of parameters. The random-effects model $\mathcal {M}_1$ is ranked with the conditional Akaike information criterion (cAIC) [33] defined as

12 $$\begin{aligned} cAIC(\mathcal {M}_1) = -2\ell (\Delta \pmb {y} \vert \pmb {u}; \pmb {\psi }) + 2(\rho + 1)\, , \end{aligned}$$

where $\ell (\Delta \pmb {y} \vert \pmb {u}; \pmb {\psi })$ is the conditional log-likelihood of the response measurements $\Delta \pmb {y}$ given the random-effects $\pmb {u}$, $\pmb {\psi }$ is the vector of all the unknown parameters, and $\rho$ is the effective degrees of freedom of $\mathcal {M}_1$ [34] defined as the trace $\rho = \text{tr}(\pmb{H})$ of the hat matrix

13 $$\begin{aligned} \pmb {H} = \begin{bmatrix} \pmb {M}&\pmb {\mathbb {M}} \end{bmatrix} \begin{bmatrix} \pmb {M}'\pmb {\Sigma }^{-1}(\pmb {\theta }, \sigma ^2)\pmb {M} &{} \pmb {M}'\pmb {\Sigma }^{-1}(\pmb {\theta }, \sigma ^2)\pmb {\mathbb {M}} \\ \pmb {\mathbb {M}}'\pmb {\Sigma }^{-1}(\pmb {\theta }, \sigma ^2)\pmb {M} &{} \pmb {\mathbb {M}}'\pmb {\Sigma }^{-1}(\pmb {\theta }, \sigma ^2)\pmb {\mathbb {M}} + \pmb {\Delta }_{\pmb {u}}^{-1} \end{bmatrix} \begin{bmatrix} \pmb {M}'\pmb {\Sigma }^{-1}(\pmb {\theta }, \sigma ^2) \\ \pmb {\mathbb {M}}'\pmb {\Sigma }^{-1}(\pmb {\theta }, \sigma ^2) \end{bmatrix}. \end{aligned}$$

To measure the distance of the fixed-effects model $\mathcal {M}_0$ from the mixed-effects model $\mathcal {M}_1$ we use the the Kullback-Leibler (KL) divergence [35]

14 $$\begin{aligned} \begin{aligned} KL_{div}(\mathcal {M}_0 \Vert \mathcal {M}_1)&= \int p(\Delta \pmb {y}) \text{ log } \frac{p(\Delta \pmb {y})}{q(\Delta \pmb {y})} d(\Delta \pmb {y}) \\&= \frac{1}{2}\left\{ \text{ tr }(\pmb {\Sigma }_1^{-1}\pmb {\Sigma }_0) - d + (\pmb {\mu }_1 - \pmb {\mu }_0)'\pmb {\Sigma }_1^{-1}(\pmb {\mu }_1 - \pmb {\mu }_0) + \text{ log }\frac{\vert \pmb {\Sigma }_1 \vert }{\vert \pmb {\Sigma }_0 \vert } \right\} \, , \end{aligned} \end{aligned}$$

where *p* and *q* are the multivariate Gaussian density functions of Eqs. (6) and (8), whose mean vector and covariance matrix are given by

15 $$\begin{aligned} &\pmb {\mu }_0 = \pmb {M} \hat{\pmb {\theta }}_{0}\, , \quad \pmb {\Sigma }_0 = \pmb {\Sigma }(\hat{\pmb {\theta }}_{0}, \hat{\sigma}^2_{0})\, , \\&\pmb {\mu }_1 = \pmb {M} \hat{\pmb {\theta }}_{1} + \pmb {\mathbb {M}}E_{\pmb {u}\vert \Delta \pmb {y}; \hat{\pmb {\psi }}}[\pmb {u}]\, , \quad \pmb {\Sigma }_1 = \pmb {\Sigma }(\hat{\pmb {\theta }}_{1}, \hat{\sigma}^2_{1})\, , \end{aligned}$$

where $(\hat{\pmb {\theta }}_{0}, \hat{\sigma}^2_{0})$ and $(\hat{\pmb {\theta }}_{1}, \hat{\sigma}^2_{1})$ are the parameter estimates for $\mathcal {M}_0$ and $\mathcal {M}_1$. To make model divergences comparable across different sized samples, we use the rescaled KL divergence $KL_{div}(\mathcal {M}_0 \Vert \mathcal {M}_1)/d$.

## Results

### In silico validation study

**Fig. 2:**
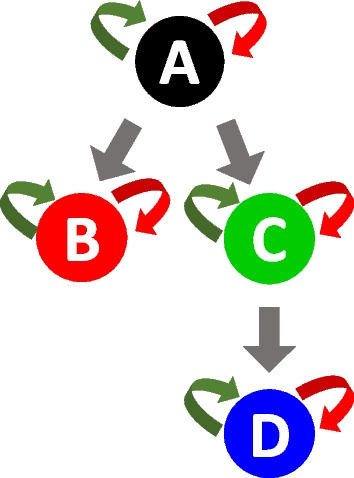
Differentiation structure of four synthetic cell types A, B, C, D. Cell duplication, cell death and cell differentiation are indicated with green, red and grey arrows

We simulated the dynamics of $J = 3$ distinct clones in four synthetic cell types A, B, C, D following the differentiation network structure of Fig. 2. The net-effect matrix $\pmb {V}$ and the hazard vector $h(\pmb {x},\pmb {\theta })$ were derived from Eq. (2). To simulate the clonal tracking data we used the $\tau$-leaping Algorithm 1 of the Additional file 1, with a time lag $\tau = 1$, that has been run independently for each clone. We designed each simulation so that the first clone dominates lineage D and the third clone dominates lineage C with a sampling frequency $T = 100$. The values that were used for the reaction parameters are reported in Table 1.

**Table 1:** For each synthetic clone (row) the parameter values (columns) used in the synthetic studies

	$\alpha _A$	$\alpha _B$	$\alpha _C$	$\alpha _D$	$\delta _A$	$\delta _B$	$\delta _C$	$\delta _D$	$\lambda _{A \rightarrow B}$	$\lambda _{A \rightarrow C}$	$\lambda _{C \rightarrow D}$
$c_1$	0.2	0.15	0.17	0.45	0.001	0.007	0.004	0.002	0.13	0.15	0.08
$c_2$	0.2	0.15	0.17	0.09	0.001	0.007	0.004	0.002	0.13	0.15	0.08
$c_3$	0.2	0.15	0.51	0.09	0.001	0.007	0.004	0.002	0.13	0.15	0.08

**Fig. 3:**
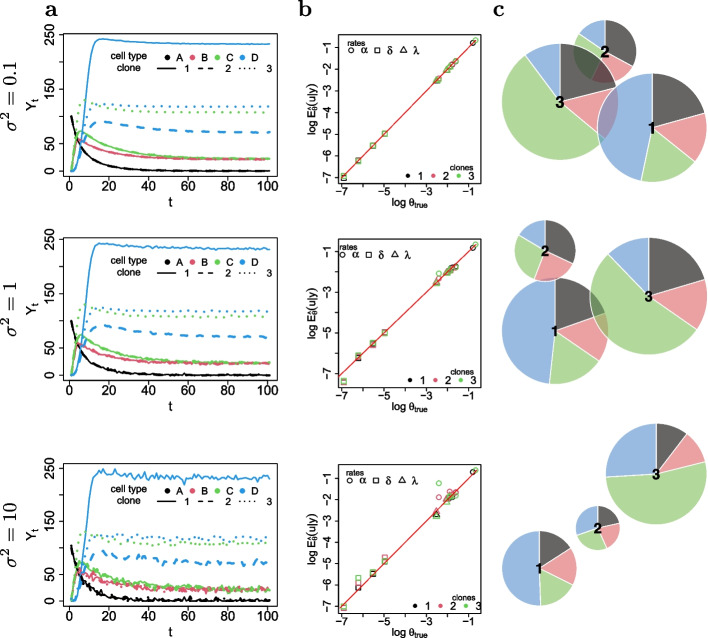
**a** Simulated trajectories. **b** Scatterplot between the clone-specific true parameters $\pmb{\theta} _{true}$ and the conditional expectation $E_{\pmb {u}\vert \Delta \pmb {y}; \hat{\pmb {\psi }}}[\pmb {u}]$. **c** Clonal pie-charts where each clone *k* is identified with a pie whose slices are lineage-specific and weighted according to Eq. (16). The diameter of the *k*-th pie is proportional to the Euclidean 2-norm of $\pmb {w}_k$, as defined in Eq. (17). Each row refers to specific values of synthetic noise variance $\sigma ^2$

**Fig. 4:**
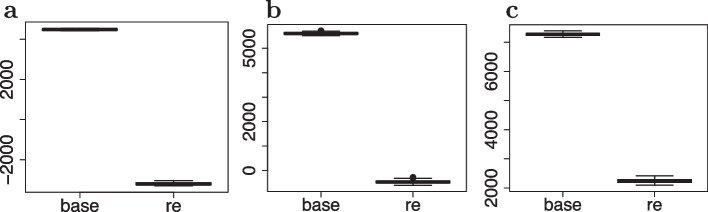
Boxplot of the AICs of the fixed-effects (base) and random-effects (re) models under a measurement noise level equal to 0.1 (**a**), 1 (**b**) and 10 (**c**)

We first ran a single simulation under different magnitudes for the noise variance $\sigma ^2$. Then we fit the random-effects model of Eq. (8) to the simulated data using Algorithm 3 from Additional file 1. We reported in Fig. 3 the simulated trajectories and a scatterplot of the estimated conditional expectation $E_{\pmb {u}\vert \Delta \pmb {y}; \hat{\pmb {\psi }}}[\pmb {u}]$ for the random-effects model against the true clone-specific parameters. In the same figure we also reported a piechart where each clone *k* is identified with a pie whose slices are lineage-specific and weighted with $w^{l}_k$, defined as the difference between the conditional expectations of the duplication and death parameters, that is

16 $$\begin{aligned} w^{l}_k = E_{\pmb {u}\vert \Delta \pmb {y}; \hat{\pmb {\psi }}}[u^k_{\alpha _{l}}] - E_{\pmb {u}\vert \Delta \pmb {y}; \hat{\pmb {\psi }}}[u^k_{\delta _{l}}]\, , \end{aligned}$$

where $u^k_{\alpha _{l}}$ and $u^k_{\delta _{l}}$ are the random-effects for duplication and death of clone *k* in cell lineage *l*. The diameter of the *k*-th pie is proportional to the Euclidean 2-norm of

17 $$\begin{aligned} \pmb {w}_k = (w^{l_1}_k,\dots , w^{l_n}_k)\, , \end{aligned}$$

where *n* is the number of cell types. Therefore, the larger the diameter, the more the corresponding clone expanded into the lineage associated to the largest slice. The values of the estimated conditional expectations are reported in Table 2. The scatterplot of Fig. 3 clearly indicates a strong agreement between the true parameters and the conditional expectations $E_{\pmb {u}\vert \Delta \pmb {y}; \hat{\pmb {\psi }}}[\pmb {u}]$. In particular, as expected, as the noise variance $\sigma ^2$ increased, the parameter estimates gradually moved away from the diagonal, so that the precision decreased. Also, our model correctly detected the dominance of clones 1 and 3 in lineages D and C respectively, even for large values of $\sigma ^2$, as suggested by the pie-charts of Fig. 3 and by the values of Table 2.

Subsequently, to check goodness-of-fit, we ran 100 independent simulations separately for each noise variance setting. After fitting both the base model of Eq. (6) and the random-effects model of Eq. (8), using Algorithms 2 and 3 of the Additional file 1, the latter always reached a significantly lower AIC compared to the null model, as suggested by the boxplots of Fig. 4. This result clearly indicates that our proposed random-effects stochastic reaction network was able to measure variation between clones in terms of differentiation dynamics and to detect events of clonal dominance.

**Table 2:** Conditional expectations $E_{\pmb {u}\vert \Delta \pmb {y}; \hat{\pmb {\psi }}}[\pmb {u}]$ of the random-effects obtained from the estimated parameters $\hat{\pmb {\psi }}$ for each reaction rate (rows) under different magnitudes of the noice variance $\sigma ^2$ (outer columns) for each clone (inner columns)

	$\sigma ^2 = 0.1$			$\sigma ^2 = 1$			$\sigma ^2 = 10$		
	$c_1$	$c_2$	$c_3$	$c_1$	$c_2$	$c_3$	$c_1$	$c_2$	$c_3$
$\alpha _{A}$	0.198	0.198	0.199	0.183	0.191	0.198	0.151	0.139	0.127
$\alpha _{B}$	0.151	0.152	0.148	0.146	0.148	0.145	0.163	0.148	0.137
$\alpha _{C}$	0.171	0.168	0.509	0.163	0.168	0.518	0.166	0.175	0.649
$\alpha _{D}$	0.446	0.094	0.098	0.450	0.100	0.121	0.479	0.199	0.319
$\delta {A}$	0.001	0.001	0.001	0.001	0.001	0.001	0.001	0.000	0.001
$\delta {B}$	0.007	0.007	0.007	0.007	0.007	0.007	0.008	0.007	0.007
$\delta {C}$	0.004	0.004	0.004	0.004	0.004	0.004	0.004	0.005	0.005
$\delta {D}$	0.002	0.002	0.002	0.002	0.002	0.002	0.002	0.003	0.004
$\delta _{A\rightarrow B}$	0.129	0.130	0.130	0.129	0.130	0.133	0.127	0.126	0.110
$\delta _{A\rightarrow C}$	0.149	0.150	0.148	0.148	0.149	0.151	0.154	0.155	0.153
$\delta _{C\rightarrow D}$	0.081	0.079	0.079	0.079	0.080	0.078	0.082	0.079	0.058

### Comparison with GLS method

**Fig. 5:**
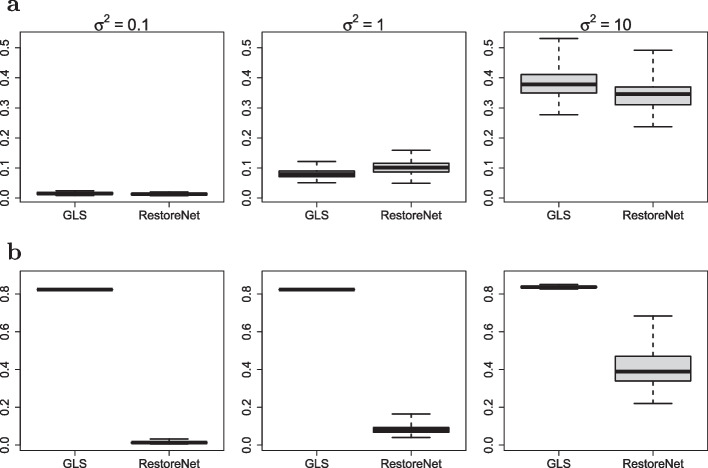
Boxplots of the relative errors between the true parameters and the estimated parameters provided by each candidate method (*x*-axis) for simulation study 1 (**a**) and 2 (**b**) under each noise variance setting (columns)

We compared our proposed method with the state-of-the-art method GLS [27]. To this end, we have designed two different simulation studies. In the first simulation study all the clones shared the same vector parameter, while in the second study we induced the same clonal expansions of previous section. In both studies we used the differentiation network structure of Fig. 2 as the true generative model from which we simulated clonal trajectories, using the $\tau$-leaping Algorithm 1 of the Additional file 1, with a time lag $\tau = 1$. The net-effect matrix $\pmb {V}$ and the hazard vector $h(\pmb {x},\pmb {\theta })$ were derived from Eq. (2). For each simulation, we ran 100 independent simulations under different noise variance settings $(\sigma ^2 \in \{0.1, 1, 10\})$. Subsequently we fit both our proposed method RestoreNet and the competitor method GLS. We reported the results in Fig. 5, showing boxplots of the relative errors between the true parameters and the estimated parameters provided by each method.

Figure 5 clearly indicates that our proposed inference method RestoreNet overall outperformed the competitor method GLS. Indeed, while in the first simulation study (no clonal dominance) both methods provided similar parameter estimates, in the second simulation study (with clonal dominance) our proposed method RestoreNet provided better parameter estimates compared to GLS. This result suggests that our proposed method RestoreNet was able to infer a cell differentiation network with clone-specific parameters. In conclusion, results from this synthetic study show that our method outperformed the competitor one for the identification of clonal dominance.

### Clonal dynamics in rhesus macaques

We analysed the cellular barcode data collected from an established hematopoietic stem cell model, previously used to investigate hematopoietic reconstitution in rhesus macaques [23]. Mobilized peripheral blood (MPB) CD34+ cells from three macaques were transduced with barcoded vectors and, following engraftment, myeloid Granulocytes (G), Monocytes (M), and lymphoid T, B, and Natural Killer (NK) cells were flow sorted for 9.5 months (ZH33), 6.5 months (ZH17), and 4.5 months (ZG66) [36]. The total numbers of clones collected are 1165 (ZH33), 1280 (ZH17), and 1291(ZG66). Further details on transduction protocols and culture conditions can be found in the original study.

Although the sample DNA amount was maintained constant during the whole experiment (200 ng for ZH33 and ZG66 or 500 ng for ZH17), the sample collected resulted in different magnitudes of total number of reads (see Table 2 from Additional file 1). This discrepancy made all the samples not directly comparable. Therefore we rescaled the barcode counts according to Eq. (34) of the Additional file 1 before analysis. We compared the base and the random-effects models on the rhesus macaques clonal tracking data. Since the CD34+ cells were not collected, we only estimated the duplication parameters $\alpha _{T}$, $\alpha _{B}$, $\alpha _{NK}$, $\alpha _{M}$, $\alpha _{G}$ and the death parameters $\delta _{T}$, $\delta _{B}$, $\delta _{NK}$, $\delta _{M}$, $\delta _{G}$ of the lymphoid (T, B, NK) and myeloid (M, G) cells. Therefore the differentiation parameters were not considered in our model, and the net-effect matrix and the hazard vector were obtained from Eqs. (2)–(5) accordingly. Thus, the biochemical reactions were defined as

18 $$\begin{aligned} \begin{aligned} x_T \overset{\alpha _{T}}{\rightarrow } 2 \cdot x_T\, , \qquad x_T \overset{\delta _{T}}{\rightarrow } \emptyset \, , \\ x_B \overset{\alpha _{B}}{\rightarrow } 2 \cdot x_B\, , \qquad x_B \overset{\delta _{B}}{\rightarrow } \emptyset \, , \\ x_{NK} \overset{\alpha _{NK}}{\rightarrow } 2 \cdot x_{NK}\, , \qquad x_{NK} \overset{\delta _{NK}}{\rightarrow } \emptyset \, , \\ x_M \overset{\alpha _{M}}{\rightarrow } 2 \cdot x_M\, , \qquad x_M \overset{\delta _{M}}{\rightarrow } \emptyset \, , \\ x_G \overset{\alpha _{G}}{\rightarrow } 2 \cdot x_G\, , \qquad x_G \overset{\delta _{G}}{\rightarrow } \emptyset \, , \end{aligned} \end{aligned}$$

where the left and right columns list the duplication and death reactions, respectively. The corresponding model became effectively a birth/death model including 10 dynamic parameters, one duplication and death rate for each cell lineage. We fit both the fixed-effects model of Eq. (6) and the mixed-effects model of Eq. (8) separately to the data of each animal. To further remove bias, we focused our analyses on the clones that were recaptured at least 5 times. This resulted in a number of clones *J* equal to 481 (ZH33), 139 (ZH17), and 202 (ZG66), and in 6 (ZH33), 5 (ZH17), and 4 (ZG66) time points. We reported the results on model selection in Table 3, and the estimated parameters $\hat{\pmb {\psi }}$ in Table 4.

**Table 3:** Comparison between fixed-effects $\mathcal {M}_0$ and mixed-effects $\mathcal {M}_1$ models: Number of parameters (*p*), AIC, KL divergence $KL_{div}(\mathcal {M}_0 \Vert \mathcal {M}_1)$ and rescaled KL divergence $KL_{div}(\mathcal {M}_0 \Vert \mathcal {M}_1)/d$ in each rhesus macaque

		*p*	AIC	$KL_{div}(\mathcal {M}_0 \Vert \mathcal {M}_1)$	$KL_{div}(\mathcal {M}_0 \Vert \mathcal {M}_1)/d$
ZH33	$\mathcal {M}_0$	11.00	81377.27		
	$\mathcal {M}_1$	434.16	38160.15	21062.95	1.87
ZH17	$\mathcal {M}_0$	11.00	336752.11		
	$\mathcal {M}_1$	478.43	29478.05	291854802.44	114228.89
ZG66	$\mathcal {M}_0$	11.00	31194.60		
	$\mathcal {M}_1$	410.92	21384.85	232030.37	83.77

**Table 4:** Parameter estimated for the proposed mixed-effects model: Fixed-effects ($\pmb {\theta }$) and variance ($\tau ^2$) of the random-effects for both the duplication $\alpha$ and death $\delta$ parameters for each cell lineage and each rhesus macaque

	ZH33		ZH17		ZG66	
	$\pmb {\theta }$	$\tau ^2$	$\pmb {\theta }$	$\tau ^2$	$\pmb {\theta }$	$\tau ^2$
$\alpha _{T}$	0.813	1.176	2.246	1.051	1.081	2.702
$\alpha _{B}$	0.193	0.597	6.503	4.648	0.055	0.876
$\alpha _{NK}$	0.758	2.253	2.435	2.364	1.095	1.943
$\alpha _{G}$	0.197	0.403	10.931	53.216	0.847	1.318
$\alpha _{M}$	0.360	0.547	3.298	4.256	2.198	1.800
$\delta _{T}$	0.155	0.074	0.172	0.741	0.039	0.059
$\delta _{B}$	0.102	0.059	2.159	36.268	0.006	0.051
$\delta _{NK}$	0.228	0.089	0.223	0.406	0.098	0.100
$\delta _{G}$	0.039	0.029	13.211	70.756	0.018	0.017
$\delta _{M}$	0.100	0.059	0.012	0.018	0.035	0.019

**Fig. 6:**
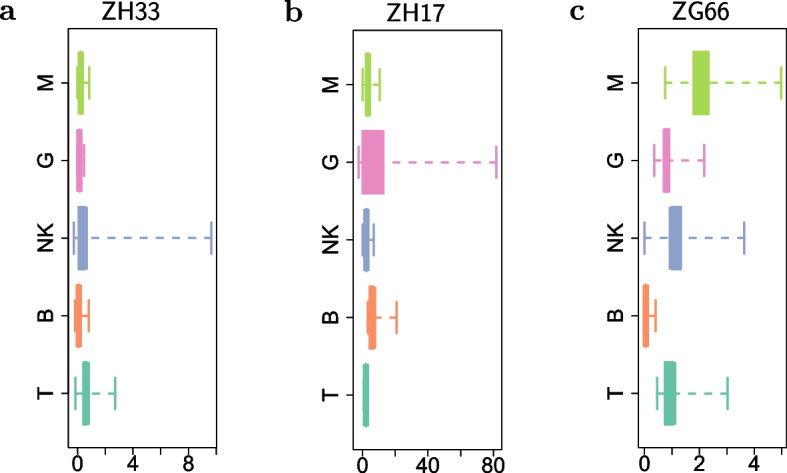
For each animal analyzed (**a**–**c**), the boxplots of the conditional expectations $E_{\pmb {u}\vert \Delta \pmb {y}; \hat{\pmb {\psi }}}[u^k_{\alpha _{l}}] - E_{\pmb {u}\vert \Delta \pmb {y}; \hat{\pmb {\psi }}}[u^k_{\delta _{l}}]$ computed from the estimated parameters $\hat{\pmb {\psi }}$ for the clone-specific net-duplication $\alpha _l - \delta _l$ in each cell lineage *l* (different colors). The whiskers extend to the data extremes

**Fig. 7:**
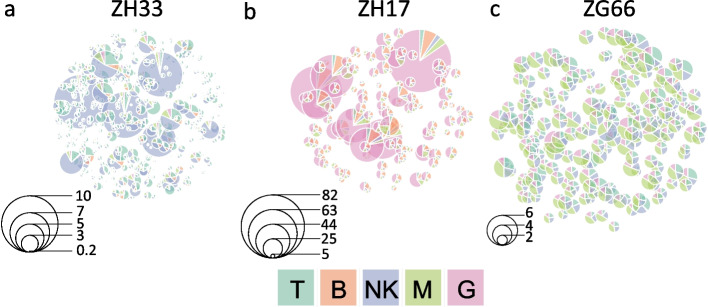
Estimated clonal pie-charts for the rhesus macaques ZH33 (**a**), ZH17 (**b**) and ZG66 (**c**): Each *k*-th clone is identified with a pie whose slices are lineage-specific and weighted according to Eq. (16). The diameter of the *k*-th pie is proportional to the Euclidean 2-norm of $\pmb {w}_k$, as defined in Eq. (17). The legend scales are different across the three plot panels

Using the estimated parameters $\hat{\pmb {\psi }}$, following Eq. (10), we computed the net conditional expectations of Eq. (16), which we used as a proxy for the clone-specific net-duplication $\alpha _l - \delta _l$ in each cell lineage *l*. The resulting values are reported in Fig. 6 in a box-plot fashion. Subsequenty, in Fig. 7 we proposed to use a weighted pie chart to visualize our findings at clonal level. Consistently with previous section, each pie, corresponding to a particular clone, was weighted by its net conditional expectations, as defined in Eq. (16).

As a result, according to the AIC values, in each animal the mixed-effects model ($\mathcal {M}_1$) outperformed the fixed-effects one ($\mathcal {M}_0$). This means that the clones did not follow the same average dynamics for the birth/death process. Instead, the dynamic of some clones departed from the average dynamics with a significant (random) effect. In particular, the conditional net-duplication rates $E_{\pmb {u}\vert \Delta \pmb {y}; \hat{\pmb {\psi }}}[u^k_{\alpha _{l}}] - E_{\pmb {u}\vert \Delta \pmb {y}; \hat{\pmb {\psi }}}[u^k_{\delta _{l}}]$ of Figs. 6 - 7 suggest events of clonal dominance in specific cell lineages. As an example, for the animals ZH33 and ZG66 we observed clonal expansions into NK cells. Whereas, for the animal ZH17 we observed clonal expansions into G and B cell lineages. Finally, for the animal ZG66 we also observed events of clonal dominance into M and T cell lineages. Furthermore, the weighted pie charts from Fig. 7 revealed different gradients of clonal dominance between the three rhesus macaques. As an example, by looking at the size of the pies, it is possible to observe an higher clonal dominance of NK cells in ZH33, and of G cells in ZH17, compared to the expansions of M, NK and T cells detected in ZG66, where the diameters of the clone-specific pies are rather similar. Not only the proposed mixed-effects model detected clonal dominance in certain cell types, it also detected which clones were responsible.

### Genotoxic effects on clonal dynamics

We analyzed an in-vivo clonal tracking dataset previously used in [30] to investigate clonal diversity in tumor-prone mice under two different treatment conditions. $Cdkn2a^{-/-}$ tumor prone $Lin^-$ cells were ex-vivo transduced with a lentiviral vector expressing GFP under either spleen focus-forming virus (SFV) or PGK promoter/enhancer sequence. Cells are then transplanted into lethally irradiated wild-type mice. To recover enough DNA material, equal amounts of blood from two or three mice belonging to the same experimental group were pooled before cell sorting. Integration sites were then retrieved by polymerase chain reaction (PCR) at different time points from sorted T (CD3+) and B (CD19+) lymphocytes, from myeloid cells (CD11b+) and unsorted blood cells (total MNC). Clonal tracking samples were collected under heterogeneous technical conditions as reported in Table 2 of the Additional file 1. These confounding effects made the samples not directly comparable. Therefore we rescaled the samples following the description in Section 2.2 of the Additional file 1 before analysis. The total number of distinct clones collected were 45186 and 20471 for the PGK and SFV treatments respectively. To further remove bias, we focused our analyses on the top $J = 1000$ most recaptured clones across lineages and time. The number of time-points *T* was equal to 7 (PGK) and 6 (SFV).

Next, we compared the fixed-effects model of Eq. (6) and the random-effects model of Eq. (8) on the rescaled clonal tracking data, so as to compare the dynamics of clonal dominance under the two viral vector conditions. Since the HSCs were not collected, we only estimated the duplication parameters $\alpha _{T}$, $\alpha _{B}$, $\alpha _{M}$ and the death parameters $\delta _{T}$, $\delta _{B}$, $\delta _{M}$ of the lymphoid (T, B) and myeloid (M) cells. Therefore, in analogy to the previous section the differentiation parameters were not considered in our model, and the net-effect matrix and the hazard vector were obtained from Eqs. (2)–(5) accordingly. Therefore, the biochemical reactions were defined as

19 $$\begin{aligned} \begin{aligned} x_T \overset{\alpha _{T}}{\rightarrow } 2 \cdot x_T\, , \qquad x_T \overset{\delta _{T}}{\rightarrow } \emptyset \, , \\ x_B \overset{\alpha _{B}}{\rightarrow } 2 \cdot x_B\, , \qquad x_B \overset{\delta _{B}}{\rightarrow } \emptyset \, , \\ x_M \overset{\alpha _{M}}{\rightarrow } 2 \cdot x_M\, , \qquad x_M \overset{\delta _{M}}{\rightarrow } \emptyset \, , \end{aligned} \end{aligned}$$

where the left and right columns list the duplication and death reactions, respectively. We fit both the fixed-effects model of Eq. (6) and the mixed-effects model of Eq. (8) separately to the data of each vector treatment. Both models included six dynamic parameters, that is one scalar value for each combination of cell type with duplication and death reactions. We reported the results on model selection in Table 5, and the estimated parameters $\hat{\pmb {\psi }}$ in Table 6.

**Table 5:** Comparison between fixed-effects $\mathcal {M}_0$ and mixed-effects $\mathcal {M}_1$ models: Number of parameters (*p*), AIC, KL divergence $KL_{div}(\mathcal {M}_0 \Vert \mathcal {M}_1)$ and rescaled KL divergence $KL_{div}(\mathcal {M}_0 \Vert \mathcal {M}_1)/d$ in each treatment group

		*p*	AIC	$KL_{div}(\mathcal {M}_0 \Vert \mathcal {M}_1)$	$KL_{div}(\mathcal {M}_0 \Vert \mathcal {M}_1)/d$
PGK	$\mathcal {M}_0$	7.00	115997.43		
	$\mathcal {M}_1$	471.40	65083.07	17098.71	1.29
SFV	$\mathcal {M}_0$	7.00	63520.89		
	$\mathcal {M}_1$	842.00	30147.56	52431.53	6.51

**Table 6:** Parameter estimated for the proposed mixed-effects model: Fixed-effects ($\pmb {\theta }$) and variance ($\tau ^2$) of the random-effects for both the duplication $\alpha$ and death $\delta$ parameters for each cell lineage and each treatment group

	PGK		SFV	
	$\pmb {\theta }$	$\tau ^2$	$\pmb {\theta }$	$\tau ^2$
$\alpha _{M}$	0.058	1.014	1.287	5.781
$\alpha _{B}$	0.092	0.872	0.024	0.408
$\alpha _{T}$	0.632	2.625	3.367	2.824
$\delta _{M}$	0.095	0.041	0.232	0.085
$\delta _{B}$	0.079	0.028	0.156	0.080
$\delta _{T}$	0.127	0.044	0.437	0.193

**Fig. 8:**
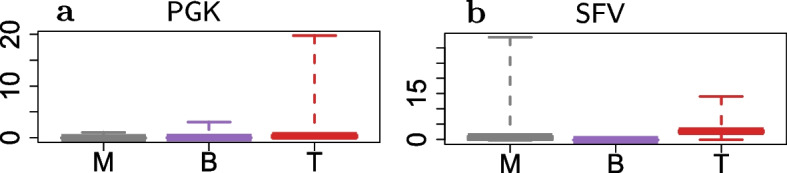
For each treatment group (**a**, **b**), the boxplots of the conditional expectations of Eq. (16) computed from the estimated parameters $\hat{\pmb {\psi }}$ for the clone-specific net-duplication $\alpha _l - \delta _l$ in each cell lineage *l* (different colors). The whiskers extend to the data extremes

**Fig. 9:**
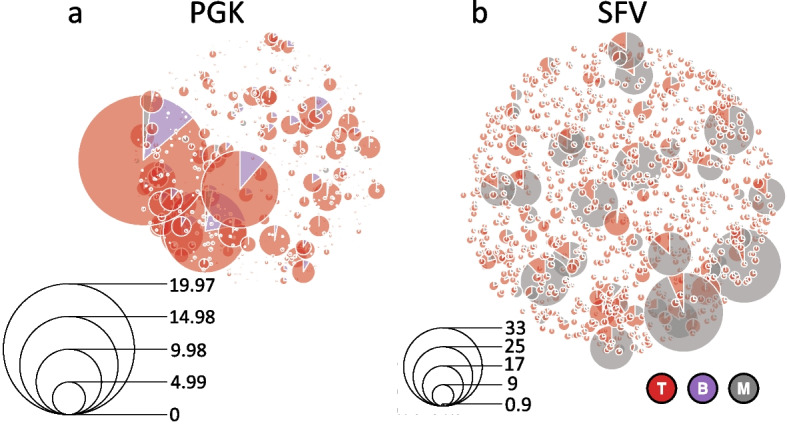
Estimated clonal pie-charts under the vector treatments PGK (**a**) and SFV (**b**): Each *k*-th clone is identified with a pie whose slices are lineage-specific and weighted according to Eq. (16). The diameter of the *k*-th pie is proportional to the Euclidean 2-norm of $\pmb {w}_k$, as defined in Eq. (17). The legend scales are different across the two plot panels

Then, from the estimated parameters $\hat{\pmb {\psi }}$ we computed the conditional expectations of Eq. (16), which we used as a proxy for the clone-specific net-duplication $\alpha _l - \delta _l$ in each cell lineage *l*. In analogy to the previous section, the resulting values are reported in Fig. 8 in a box-plot fashion, while in Fig. 9 we proposed to use a weighted pie chart to visualize our findings at clonal level.

As a result, according to the AIC values, under each treatment the mixed-effects model ($\mathcal {M}_1$) outperformed the fixed-effects one ($\mathcal {M}_0$). This means that the clones exhibited heterogeneity in their dynamics for the birth/death process. The dynamics of some clones departed from the average dynamics with a significant (random) effect. In particular, the conditional net-duplication rates of Eq. (16) from Figs. 8 - 9 suggest events of clonal dominance in specific cell lineages. For example, under the PGK treatment we observed clonal expansions into T cells. Whereas, under the SFV treatment we observed clonal expansions into M and T cell lineages with even higher conditional rates compared to PGK. Furthermore, the Kullback–Leibler divergence from Table 5 revealed a different gradient of clonal dominance between the two treatments, suggesting that the clonal expansions identified in the SFV case were more significant compared to PGK.

## Discussion and conclusion

In this work we proposed a random-effects cell differentiation network which takes into account heterogeneity in the dynamics across the clones. Our framework extends the clone neutral local linear approximation of a stochastic quasi-reaction network, written in the Ito formulation, by introducing random-effects for the clones on the dynamics parameters to allow for clonal dominance. We used a maximum likelihood approach to infer the parameters of the base (fixed-effects only) model that are than used as initial values for the estimation of the random-effects model by means of an E-M algorithm. We tested our framework with a $\tau$-leaping simulation study, showing accurate performance of the method in the identification of a clonal expansion and in the inference of the true parameters. Then, by means of an additional in-silico study, we have shown that our method outperforms the state-of-the-art method GLS [27]. Subsequently, the application of our proposed method on a rhesus macaque clonal tracking study revealed significant clonal dominance for specific cell types. Particularly interesting is that the NK clonal expansions detected by our model were already observed by former studies [23, 37, 38], and therefore our findings are consistent with those previously obtained. Indeed [37] described the oligoclonal expansions of NK cells and the long-term persistence of HSPCs and immature NK cells. Finally, our proposed method allowed to detect the expected impact of vector genotoxicity on clonal dynamics in a tumor-prone mice model of haematopoiesis, as already observed in a previous study [30].

The main approximation, in both the basal and random-effects formulations, is the piece-wise linearity of the process. In both cases we consider first a local linear approximation of the Kramers-Moyal approximated Master equation, which is then used to infer the process parameters either with or without random-effects. Although the linearity assumption makes all the computations easier, this approximation becomes poor as the time lag increments (the $\Delta t$s) of the collected data increase. This can be addressed by introducing in the likelihood higher-order approximation terms than the ones considered by the Euler-Maruyama method. The Milstein approximation is a possible choice [39]. Another, completely different, approach is to employ extended Kalman filtering (EKF) which is suitable for non-linear state space formulations [40]. Also, our framework cannot consider false-negative errors or missing values of clonal tracking data. Also for this issue, an EKF formulation could be a possible extension. The frequentist-based inference step of our proposed E-M algorithm may be replaced by Bayesian alternatives. For example, the E-step function $Q(\pmb {\psi }\vert \pmb {\psi }^*)$ could be replaced by a Metropolis-Hastings step [41, 42]. Alternatively, a variational Bayes method could be employed, where the unknown vector parameter $\pmb {\psi }$ is treated as an additional latent variable [43]. Our future work will aim to extend the 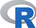 package RestoreNet by including other types of reactions (besides cell duplication, cell death and cell differentiation).

Our tool can be considered as complementary to the classical Shannon entropy index [30] in detecting fast and uncontrolled growing of clones after a gene therapy treatment. Indeed, while the Shannon entropy measures the diversity of a population of clones as a whole, RestoreNet provides a clone-specific quantification of dominance in terms of conditional mean and variance of the expansion rates. Our proposed method provides a prototype model of clonal haematopoiesis whose parameters are calibrated to fit high-dimensional clonal tracking data. Our data-driven model can be integrated with those obtained with alternative approaches, where the unknown parameters are either set to experimentally-derived quantities, computed from the steady states, or based on independent studies [44, 45].

In conclusion, our proposed stochastic framework is able to detect deviant clonal behaviour relative to the average dynamics of haematopoiesis. This is an important aspect for gene therapy applications where is crucial to quickly detect any adverse event that may be related to clonal dominance. Therefore our tool can provide statistical support in gene therapy surveillance analyses. Our proposed method also has potential applications in other biomedical longitudinal studies with subject-specific dynamics, such as population infection dynamics [46, 47], population analysis of tumor development [48], and genetic regulatory networks [49]. Moreover, our proposed mixed-effects formulation of stochastic quasi-reaction networks can potentially be applied to more general, non-Markovian, classes of network models, such as stochastic hybrid systems with memory (SHSM). This more general class of models suits history-dependent biological systems, such as neural dynamics and immune responses [50, 51]. A mixed-effects formulation of dynamical systems may find room also in optimal investments problems, such as stochastic games in a continuous-time Markov regime-switching environment [52]. Indeed, if such models can be written in a Ito-type formulation, mixed-effects on sensible subjects (e.g. groups of investors in a market) can be incorporated.

## Supplementary Information

**Additional file 1.** Detailed description of the methods

## Data Availability

All data analysed in this work are available from earlier publications [23, 30]. The code that supports the findings of this study is openly available at https://github.com/delcore-luca/ClonalDominance. The stochastic framework is implemented in the 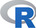 package RestoreNet publicly available for download at https://CRAN.R-project.org/package=RestoreNet.

## References

[1] Friedmann T, Roblin R (1972). Gene therapy for human genetic disease?. Science.

[2] Bryder D, Rossi DJ, Weissman IL (2006). Hematopoietic stem cells: the paradigmatic tissue-specific stem cell. Am J Pathol.

[3] Kustikova OS, Wahlers A, Kühlcke K, Stähle B, Zander AR, Baum C, Fehse B (2003). Dose finding with retroviral vectors: correlation of retroviral vector copy numbers in single cells with gene transfer efficiency in a cell population. Blood.

[4] Fehse B, Kustikova O, Bubenheim M, Baum C (2004). Pois (s) on-it’s a question of dose..... Gene Ther.

[5] Baum C, Düllmann J, Li Z, Fehse B, Meyer J, Williams DA, Von Kalle C (2003). Side effects of retroviral gene transfer into hematopoietic stem cells. Blood J Am Soc Hematol.

[6] Modlich U, Kustikova OS, Schmidt M, Rudolph C, Meyer J, Li Z, Kamino K, Von Neuhoff N, Schlegelberger B, Kuehlcke K (2005). Leukemias following retroviral transfer of multidrug resistance 1 (mdr1) are driven by combinatorial insertional mutagenesis. Blood.

[7] Baum C, Kustikova O, Modlich U, Li Z, Fehse B (2006). Mutagenesis and oncogenesis by chromosomal insertion of gene transfer vectors. Hum Gene Ther.

[8] Catlin SN, Guttorp P, Abkowitz JL (2005). The kinetics of clonal dominance in myeloproliferative disorders. Blood.

[9] Roeder I, Horn M, Glauche I, Hochhaus A, Mueller MC, Loeffler M (2006). Dynamic modeling of imatinib-treated chronic myeloid leukemia: functional insights and clinical implications. Nat Med.

[10] Müller-Sieburg CE, Cho RH, Thoman M, Adkins B, Sieburg HB (2002). Deterministic regulation of hematopoietic stem cell self-renewal and differentiation. Blood J Am Soc Hematol.

[11] Roeder I, Kamminga LM, Braesel K, Dontje B, de Haan G, Loeffler M (2005). Competitive clonal hematopoiesis in mouse chimeras explained by a stochastic model of stem cell organization. Blood.

[12] Sieburg HB, Cho RH, Dykstra B, Uchida N, Eaves CJ, Muller-Sieburg CE (2006). The hematopoietic stem compartment consists of a limited number of discrete stem cell subsets. Blood.

[13] Loeffler M, Birke A, Winton D, Potten C (1993). Somatic mutation, monoclonality and stochastic models of stem cell organization in the intestinal crypt. J Theor Biol.

[14] Loeffler M, Bratke T, Paulus U, Li Y, Potten C (1997). Clonality and life cycles of intestinal crypts explained by a state dependent stochastic model of epithelial stem cell organization. J Theor Biol.

[15] Loeffler M, Roeder I (2002). Tissue stem cells: definition, plasticity, heterogeneity, self-organization and models-a conceptual approach. Cells Tissues Organs.

[16] Meineke FA, Potten CS, Loeffler M (2001). Cell migration and organization in the intestinal crypt using a lattice-free model. Cell Prolif.

[17] Roeder I, Braesel K, Lorenz R, Loeffler M. Stem cell fate analysis revisited: interpretation of individual clone dynamics in the light of a new paradigm of stem cell organization. J Biomed Biotechnol. 2007;2007. 10.1155/2007/84656 PMC1874676 17541472

[18] Winton D, Blount M, Ponder B (1988). A clonal marker induced by mutation in mouse intestinal epithelium. Nature.

[19] Park H-S, Goodlad RA, Wright NA (1995). Crypt fission in the small intestine and colon. A mechanism for the emergence of g6pd locus-mutated crypts after treatment with mutagens. Am J Pathol.

[20] Bjerknes M, Cheng H (2001). Modulation of specific intestinal epithelial progenitors by enteric neurons. Proc Natl Acad Sci.

[21] Potten CS, Booth C, Pritchard DM (1997). The intestinal epithelial stem cell: the mucosal governor. Int J Exp Pathol.

[22] Biasco L, Pellin D, Scala S, Dionisio F, Basso-Ricci L, Leonardelli L, Scaramuzza S, Baricordi C, Ferrua F, Cicalese M, Giannelli S, Neduva V, Dow D, Schmidt M, Von Kalle C, Roncarolo M, Ciceri F, Vicard P, Wit E, Di Serio C, Naldini L, Aiuti A (2016). In vivo tracking of human hematopoiesis reveals patterns of clonal dynamics during early and steady-state reconstitution phases. Cell Stem Cell.

[23] Wu C, Li B, Lu R, Koelle S, Yang Y, Jares A, Krouse A, Metzger M, Liang F, Loré K, Wu C, Donahue R, Chen IY, Weissman I, Dunbar C (2014). Clonal tracking of rhesus macaque hematopoiesis highlights a distinct lineage origin for natural killer cells. Cell Stem Cell.

[24] Mazurier F, Gan OI, McKenzie JL, Doedens M, Dick JE (2004). Lentivector-mediated clonal tracking reveals intrinsic heterogeneity in the human hematopoietic stem cell compartment and culture-induced stem cell impairment. Blood.

[25] Biasco L, Rothe M, Schott JW, Schambach A (2017). Integrating vectors for gene therapy and clonal tracking of engineered hematopoiesis. Hematol/Oncol Clin.

[26] Pellin D. Stochastic modelling of dynamical systems in biology [phd thesis]. PhD thesis, University of Groningen; 2017.

[27] Pellin D, Biasco L, Aiuti A, Di Serio MC, Wit EC (2019). Penalized inference of the hematopoietic cell differentiation network via high-dimensional clonal tracking. Appl Netw Sci.

[28] Bailey NTJ. The elements of stochastic processes with applications to the natural sciences. Wiley Classics Library, Wiley; 1990. https://books.google.it/books?id=yHPnwl4QOfIC.

[29] Kloeden PE, Platen E. Numerical solution of stochastic differential equations. In: Stochastic modelling and applied probability. Springer; 2011. https://books.google.it/books?id=BCvtssom1CMC.

[30] Del Core L, Cesana D, Gallina P, Secanechia YNS, Rudilosso L, Montini E, Wit EC, Calabria A, Grzegorczyk MA (2022). Normalization of clonal diversity in gene therapy studies using shape constrained splines. Sci Rep.

[31] Dobson AJ, Barnett AG. An Introduction to Generalized Linear Models. Chapman & Hall/CRC Texts in Statistical Science. CRC Press; 2018. https://books.google.it/books?id=kIhnDwAAQBAJ.

[32] Vaida F, Blanchard S (2005). Conditional Akaike Information for mixed-effects models. Biometrika.

[33] Burnham KP, Anderson DR, Huyvaert KP (2011). AIC model selection and multimodel inference in behavioral ecology: some background, observations, and comparisons. Behav Ecol Sociobiol.

[34] Müller S, Scealy JL, Welsh AH (2013). Model selection in linear mixed models. Stat Sci.

[35] Kullback S, Leibler RA (1951). On information and sufficiency. Ann Math Stat.

[36] Lu R, Neff NF, Quake SR, Weissman IL (2011). Tracking single hematopoietic stem cells in vivo using high-throughput sequencing in conjunction with viral genetic barcoding. Nat Biotechnol.

[37] Wu C, Espinoza DA, Koelle SJ, Yang D, Truitt L, Schlums H, Lafont BA, Davidson-Moncada JK, Lu R, Kaur A (2018). Clonal expansion and compartmentalized maintenance of rhesus macaque NK cell subsets. Sci Immunol.

[38] Wu C, Mortlock RD, Shin T, Cordes S, Fan X, Brenchley J, Allan DA, Hong SG, Dunbar CE (2021). Tissue-resident clonal expansions of rhesus macaque NK cells. Blood.

[39] Mil’shtejn GN (1975). Approximate integration of stochastic differential equations. Theory Probab Its Appl.

[40] Jazwinski AH. Stochastic Processes and Filtering Theory. Courier Corporation; 2007.

[41] Metropolis N, Rosenbluth AW, Rosenbluth MN, Teller AH, Teller E (1953). Equation of state calculations by fast computing machines. J Chem Phys.

[42] Hastings WK. Monte Carlo sampling methods using Markov chains and their applications; 1970.

[43] MacKay DJ, Mac Kay DJ. Information Theory. Inference and Learning Algorithms. Cambridge University Press; 2003.

[44] Ashcroft P, Manz MG, Bonhoeffer S (2017). Clonal dominance and transplantation dynamics in hematopoietic stem cell compartments. PLoS Comput Biol.

[45] Pedersen RK, Andersen M, Stiehl T, Ottesen JT (2021). Mathematical modelling of the hematopoietic stem cell-niche system: clonal dominance based on stem cell fitness. J Theor Biol.

[46] Liu D, Lu T, Niu X-F, Wu H (2011). Mixed-effects state-space models for analysis of longitudinal dynamic systems. Biometrics.

[47] Nowak MA, Bangham CRM (1996). Population dynamics of immune responses to persistent viruses. Science.

[48] Ribba B, Holford N, Magni P, Trocóniz I, Gueorguieva I, Girard P, Sarr C, Elishmereni M, Kloft C, Friberg L (2014). A review of mixed-effects models of tumor growth and effects of anticancer drug treatment used in population analysis. CPT Pharmacomet Syst Pharmacol.

[49] Schlitt T, Brazma A (2007). Current approaches to gene regulatory network modelling. BMC Bioinform.

[50] Gokgoz N, Öktem H (2021). Modeling of tumor-immune system interaction with stochastic hybrid systems with memory: a piecewise linear approach. Adv Theory Nonlinear Anal its Appl.

[51] Weber G-W, Ugur O, Taylan P, Tezel A (2009). On optimization, dynamics and uncertainty: a tutorial for gene-environment networks. Discret Appl Math.

[52] Savku E, Weber G-W (2022). Stochastic differential games for optimal investment problems in a Markov regime-switching jump-diffusion market. Ann Oper Res.

