# Supplementary material for: A mixed-effects stochastic model reveals clonal dominance in gene therapy safety studies
Source: BMC Bioinformatics. 2023 Jun 2;24:228. doi: 10.1186/s12859-023-05269-1 (PMC10239124; doi:10.1186/s12859-023-05269-1)
Supplement: Supplementary file 1 — Additional file 1. Detailed description of the methods [file 12859_2023_5269_MOESM1_ESM.pdf]

S1 Text: A mixed-effects stochastic model reveals clonal dominance in gene therapy safety studies

L. Del Core et al.
luca.delcore@nottingham.ac.uk

1 Mathematical details

1.1 Stochastic quasi-reaction networks

Stochastic quasi-reaction networks (S-QRNs) allow to implement a particular class of stochastic differential equations that can be used to model biochemical reactions. More formally, let

$$\boldsymbol{y}_t = (y_{1t}, \dots, y_{nt})' \in \mathbb{N}_0^n \tag{1}$$

be a collection of molecules of n different types observed at time t, and consider K distinct (and competing) reactions

$$r_{j1}y_1 + \dots + r_{jn}y_n \xrightarrow{\theta_j} p_{j1}y_1 + \dots + p_{jn}y_n \,, \quad j = 1, \dots, K \tag{2}$$

each occurring with its own rate θ_j. The coefficients r_{ji}'s defining the left-side of the reaction are called reagents and represent the minimum amount of molecules of type i needed for the j-th reaction to occur. Similarly, the coefficients p_{ji} defining the right-side of the reaction are called products and represent the amount of produced molecules of type i after the j-th reaction is triggered. We assume that, if we observe $\boldsymbol{y}_0 = (r_{j1}, \dots, r_{jn})'$ molecules at time $t = 0$, the j-th reaction will occur after

$$T_j \sim \text{Exp}(\theta_j) \,, \quad j = 1, \dots, K \,, \tag{3}$$

Namely, if exactly r_{ij} molecules of each type i would be present, then the j-th reaction can only take place in one way, with the exponential hazard rate θ_j. The interpretation is that, after a waiting time T_j, r_{ji} molecules of type i collide with each other and produce p_{ji} molecules of type i ($\forall i = 1, \dots, n$), while the molecules move randomly in a hosting "cellular" environment. However, in general at time $t = 0$ we might observe $Y_{i0} \geq r_{ji}$ molecules of each type i and, therefore, the j-th reaction can take place in a combinatorial number of ways leading to the following waiting time formulation

$$T_j \sim \text{Exp}\left(\theta_j \prod_{i=1}^{n} \binom{y_{i0}}{r_{ji}}\right) \,, \quad \text{where } \binom{x}{y} = 0 \,, \quad \text{for } x < y \,, \tag{4}$$

where

$$\boldsymbol{\theta} = (\theta_1, \dots, \theta_K)' \tag{5}$$

is the vector parameter for the reaction rates, and

$$h_j(\boldsymbol{y}_0, \boldsymbol{\theta}) = \theta_j \prod_{i=1}^{n} \binom{y_{i0}}{r_{ji}} \tag{6}$$

is the j-th hazard rate. In this case, the effect will be that at time $t + T_j$ we have the following expression for the number of molecules of substrate i,

$$y_{i,t+T_j} = y_{it} + p_{ji} - r_{ji} = y_{it} + v_{ji} \,, \tag{7}$$

where $v_{ji} = p_{ji} - r_{ji}$ is the j-th net effect. More compactly, for a set of K reactions and n species, the molecular transfer from reagent to product species is a net change of

$$\boldsymbol{V} = \boldsymbol{P} - \boldsymbol{R} \,, \tag{8}$$

where $\boldsymbol{P} = [p_{ji}]'$ denotes the $n \times r$ dimensional matrix of products, $\boldsymbol{R} = [r_{ji}]'$ is the $n \times r$ dimensional matrix of reactants, and $\boldsymbol{V} = [v_{ji}]'$ is an $n \times r$ dimensional matrix called ***net-effect matrix***. Therefore, a S-QRN of K-distinct reactions is fully identified by a net-effect matrix $\boldsymbol{V}$ and by the ***hazard vector***

$$\boldsymbol{h}(\boldsymbol{y}, \boldsymbol{\theta}) = (h_1(\boldsymbol{y}, \boldsymbol{\theta}), \ldots, h_K(\boldsymbol{y}, \boldsymbol{\theta}))' \,. \tag{9}$$

1.2 Simulating a trajectory of molecules

A τ-leaping algorithm is an alternative method to a Gillespie algorithm for simulating triggering-chain events. Instead of simulating a waiting time for the first reaction to occur and selecting the corresponding winner reaction, a τ-leaping algorithm simulates the number of occurrences of each possible event after a time-lag equal to τ elapsed. Formally, let $\{N_r(t)\}_{t \geq 0}$ be an inhomogeneous Poisson point process representing the number of reactions of type r that took place up to (and including) time t. Therefore

$$\begin{aligned} N_r(t) &\sim \text{Poisson}\left(\int_0^t \theta_r(s) ds\right) \,, \\ E[N_r(t + \Delta t) - N_r(t)] &= \int_t^{t+\Delta t} \theta_r(s) ds \,\widehat{=}\, \Theta_t^r \,. \end{aligned} \tag{10}$$

The last equation gives an estimate of the expected number of reactions of type r that took place within the time interval $[t, t+\Delta_t[$. Therefore, the expected number of molecules $\boldsymbol{y}_{t+\Delta t}$ at time $t + \Delta t$ given the current number of molecules $\boldsymbol{y}_t$ can be easily obtained by adding to $\boldsymbol{y}_t$ the product between the expected number of events $E[N_r(t + \Delta t) - N_r(t)]$ that have happened in the time interval $[t, t + \Delta_t[$, the corresponding net-effect, and the number of ways that reaction can occur, leading to

$$\boldsymbol{y}_{t+\Delta t} = \boldsymbol{y}_t + \boldsymbol{V} \begin{bmatrix} \Theta_t^1 \prod_{i=1}^n \binom{y_{it}}{r_{1i}} \\ \vdots \\ \Theta_t^K \prod_{i=1}^n \binom{y_{it}}{r_{Ki}} \end{bmatrix} \,. \tag{11}$$

The pseudocode of the τ-leaping algorithm (τ-LA) is reported in Algorithm 1.

1.3 The Master Equation

In practice it is common that the reaction rates of a stochastic reaction network are unknown, and the goal is to estimate them given a collected dataset. In order to estimate the rates $\boldsymbol{\theta} = (\theta_1, \ldots, \theta_K)'$ using a likelihood-based approach, we need to define an underlying probabilistic model. One of the most natural choices for describing stochastic chemical kinetics of Eqs. (2)-(9) is the ***chemical master equation***

$$\frac{dP(\boldsymbol{y};t)}{dt} = \sum_{j=1}^K \left\{ h_j(\boldsymbol{y} - V_{\cdot j}; \boldsymbol{\theta}) P(\boldsymbol{y} - V_{\cdot j}; t) - h_j(\boldsymbol{y}; \boldsymbol{\theta}) P(\boldsymbol{y}; t) \right\} \,, \tag{12}$$

Algorithm 1: τ-leaping algorithm

Input: S `(no. simulations)`, $\boldsymbol{y}_0$`(initial state)`, τ `(time lag)`, $\theta(t)$ `(reaction rates)`
Output: $\{\boldsymbol{y}_t\}_t$
$t \leftarrow 0$;
$\boldsymbol{y}_t \leftarrow \boldsymbol{y}_0$;
for $s = 1 : S$ **do**
  for $r = 1 : K$ **do**
    $\Theta_t^r = \int_t^{t+\Delta t} \theta_r(s) ds$;
  end
  $$\boldsymbol{y}_{t+\Delta t} \leftarrow \boldsymbol{y}_t + \boldsymbol{V} \begin{bmatrix} \Theta_t^1 \prod_{i=1}^n \binom{y_{it}}{r_{1i}} \\ \vdots \\ \Theta_t^K \prod_{i=1}^n \binom{y_{it}}{r_{Ki}} \end{bmatrix};$$
  $t \leftarrow t + \tau$;
end

with transition rates

$$h_j(\boldsymbol{y}; \theta) = \theta_j \prod_{i=1}^n \binom{y_i}{r_{ji}}, \tag{13}$$

consistently with Eq. (4). It describes the temporal evolution of the probability density function $P(\boldsymbol{y}; t)$ of the state vector $\boldsymbol{y}$ of the chemical system (2). Roughly speaking, the first part of the right-hand side of Eq. (12) models all the reactions letting the state out of $k(\neq j)$, whereas the second part models all the reactions which brings the state back to k. It is often the case that the Master equation is computationally intractable, especially when the state vector $\boldsymbol{y}$ is high-dimensional, so that the number of possible states the system may occupy is too large. Several approximations of the Master equation exist [1,2], and here we describe a procedure for "continuizing" the discrete-state chemical Markov process defined by Eqs. (2)-(12). The procedure is summarized in the following theorem.

Theorem 1. *Assume that $h_j(\boldsymbol{x}; \boldsymbol{\theta})P(\boldsymbol{x}; t)$ are analytical functions in $\boldsymbol{x}$. Then, a second order Taylor expansion of the products $h_j(\boldsymbol{x} - V_{\cdot j}; \boldsymbol{\theta})P(\boldsymbol{x} - V_{\cdot j}; t)$ around $\boldsymbol{x}$ leads to the Ito-type stochastic differential equation*

$$d\boldsymbol{x}_t = \mu(\boldsymbol{x}_t; \boldsymbol{\theta})dt + \beta^{1/2}(\boldsymbol{x}_t; \boldsymbol{\theta})d\boldsymbol{W}(t), \quad d\boldsymbol{W}(t) \sim N(\boldsymbol{0}, dt\boldsymbol{I}), \tag{14}$$

*called the **Kramers-Moyal approximation** where the drift function and the dispersion matrix are given by*

$$\boldsymbol{\mu}(\boldsymbol{x}_t; \boldsymbol{\theta}) = V\boldsymbol{h}(\boldsymbol{x}_t, \boldsymbol{\theta}) \tag{15}$$

$$\boldsymbol{\beta}(\boldsymbol{x}_t; \boldsymbol{\theta}) = V \underbrace{\begin{bmatrix} h_1(\boldsymbol{x}_t; \boldsymbol{\theta}) & & \\ & \ddots & \\ & & h_K(\boldsymbol{x}_t; \boldsymbol{\theta}) \end{bmatrix}}_{d(\boldsymbol{h}(\boldsymbol{x}_t, \theta))} V' \tag{16}$$

Proof. The analytical assumption of $h_j(\boldsymbol{x}; \boldsymbol{\theta})P(\boldsymbol{y}; t)$ in $\boldsymbol{x}$ allows us to consider a second-

order Taylor expansion of $h_j(\boldsymbol{x} - V_{\cdot j}; \boldsymbol{\theta})P(\boldsymbol{x} - V_{\cdot j}; t)$ around $\boldsymbol{x}$, that is

$$\begin{aligned}
&h_j(\boldsymbol{x} - V_{\cdot j}; \boldsymbol{\theta})P(\boldsymbol{x} - V_{\cdot j}; t) \\
&= h_j(\boldsymbol{x}; \boldsymbol{\theta})P(\boldsymbol{x}; t) + \nabla_{\boldsymbol{x}} h_j(\boldsymbol{x}; \boldsymbol{\theta})P(\boldsymbol{x}; t)\left((\boldsymbol{x} - V_{\cdot j}) - \boldsymbol{x}\right) \\
&+ \frac{1}{2}\left((\boldsymbol{x} - V_{\cdot j}) - \boldsymbol{x}\right)' H_{\boldsymbol{x}} h_j(\boldsymbol{x}; \boldsymbol{\theta})P(\boldsymbol{x}; t)\left((\boldsymbol{x} - V_{\cdot j}) - \boldsymbol{x}\right) \\
&= h_j(\boldsymbol{x}; \boldsymbol{\theta})P(\boldsymbol{x}; t) - \nabla_{\boldsymbol{x}}\left\{h_j(\boldsymbol{x}; \boldsymbol{\theta})P(\boldsymbol{x}; t)\right\} V_{\cdot j} + \frac{1}{2} V_{\cdot j}' H_{\boldsymbol{x}}\left\{h_j(\boldsymbol{x}; \boldsymbol{\theta})P(\boldsymbol{x}; t)\right\} V_{\cdot j}\,,
\end{aligned}$$

and therefore

$$\begin{aligned}
&h_j(\boldsymbol{x} - V_{\cdot j}; \boldsymbol{\theta})P(\boldsymbol{x} - V_{\cdot j}; t) - h_j(\boldsymbol{x}; \boldsymbol{\theta})P(\boldsymbol{x}; t) \\
&= -\nabla_{\boldsymbol{x}}\left\{h_j(\boldsymbol{x}; \boldsymbol{\theta})P(\boldsymbol{x}; t)\right\} V_{\cdot j} + \frac{1}{2} V_{\cdot j}' H_{\boldsymbol{x}}\left\{h_j(\boldsymbol{x}; \boldsymbol{\theta})P(\boldsymbol{x}; t)\right\} V_{\cdot j}\,,
\end{aligned}$$

and by plugging it in the Master equation (12) we have

$$\begin{aligned}
\frac{\partial P(\boldsymbol{x}, t)}{\partial t} &= \sum_{j=1}^{K}\left\{-\nabla_{\boldsymbol{x}}\left\{h_j(\boldsymbol{x}; \boldsymbol{\theta})P(\boldsymbol{x}; t)\right\} V_{\cdot j} + \frac{1}{2} V_{\cdot j}' H_{\boldsymbol{x}}\left\{h_j(\boldsymbol{x}; \boldsymbol{\theta})P(\boldsymbol{x}; t)\right\} V_{\cdot j}\right\} \\
&= -\nabla_{\boldsymbol{x}}\left\{\boldsymbol{V}\boldsymbol{h}(\boldsymbol{x}; \boldsymbol{\theta})P(\boldsymbol{x}; t)\right\} + \frac{1}{2}\nabla_{\boldsymbol{x}}^2\left\{\boldsymbol{V}\begin{bmatrix} h_1(\boldsymbol{x}_t; \boldsymbol{\theta}) & & \\ & \ddots & \\ & & h_K(\boldsymbol{x}_t; \boldsymbol{\theta})\end{bmatrix}\boldsymbol{V}' P(\boldsymbol{x}; t)\right\}\,,
\end{aligned}$$

which we recognize as a Kolmogorov forward (Fokker-Plank) equation with drift function $\boldsymbol{V}\boldsymbol{h}(\boldsymbol{x}; \boldsymbol{\theta})$ and dispersion matrix $\boldsymbol{V}d(\boldsymbol{h}(\boldsymbol{x}; \boldsymbol{\theta}))\boldsymbol{V}'$, which completes the proof. □

1.3.1 Euler-Maruyama approximation

Remark 1. ***(Generalized Linear Model (GLM) formulation)***

Using previous results and some linear algebra, the approximated Ito equation (14) can be further approximated as

$$\Delta \boldsymbol{y}_t = \overbrace{\boldsymbol{V}\begin{bmatrix} \prod_{i=1}^{n}\binom{y_{it}}{r_{1i}} & & \\ & \ddots & \\ & & \prod_{i=1}^{n}\binom{y_{it}}{r_{Ki}}\end{bmatrix}}^{\boldsymbol{M}_t}\Delta t \underbrace{\begin{bmatrix}\theta_1 \\ \vdots \\ \theta_K\end{bmatrix}}_{\boldsymbol{\theta}} + \left(\underbrace{\boldsymbol{V}\begin{bmatrix} h_1(\boldsymbol{y}_t, \boldsymbol{\theta}) & & \\ & \ddots & \\ & & h_1(\boldsymbol{y}_t, \boldsymbol{\theta})\end{bmatrix}\boldsymbol{V}'\Delta t}_{\boldsymbol{W}_t(\theta)} + \sigma^2 \boldsymbol{I}_n\right)^{1/2}\Delta \boldsymbol{\varepsilon}_t\,, \tag{17}$$

$$\Delta \boldsymbol{\varepsilon}_t \sim \mathscr{N}(\boldsymbol{0}, \boldsymbol{I}_n)\,,$$

or more compactly

$$\Delta \boldsymbol{y}_t = \boldsymbol{M}_t\boldsymbol{\theta} + \boldsymbol{\varepsilon}_t\,, \quad \boldsymbol{\varepsilon}_t \sim \mathscr{N}_N(\boldsymbol{0}, \boldsymbol{W}_t(\boldsymbol{\theta}) + \sigma^2 \boldsymbol{I}_n)\,, \tag{18}$$

where we included the term $\sigma^2 \boldsymbol{I}_N$ so as to prevent singularity of the diffusion term, and to additionally explain noise variance. In practice, since we collect only discrete-time increments $\Delta \boldsymbol{y}_t = \boldsymbol{y}_{t+\Delta t} - \boldsymbol{y}_t$, we consider an Euler-Maruyama local linear approximation (LLA) of the approximated Ito equation. Indeed we also replaced the infinitesimal increments dt and $d\boldsymbol{y}_t$ with the discrete increments Δt and $\Delta \boldsymbol{y}_t$. Then, all the time-specific blocks can be stacked together obtaining the full generalized linear model (GLM) formulation

$$\underbrace{\begin{bmatrix}\Delta \boldsymbol{y}_{t_0} \\ \vdots \\ \Delta \boldsymbol{y}_{t_{T-1}}\end{bmatrix}}_{\Delta \boldsymbol{y}} = \underbrace{\begin{bmatrix}\boldsymbol{M}_{t_0} \\ \vdots \\ \boldsymbol{M}_{t_{T-1}}\end{bmatrix}}_{\boldsymbol{M}}\boldsymbol{\theta} + \boldsymbol{\varepsilon}\,, \quad \boldsymbol{\varepsilon} \sim \mathscr{N}\left(\boldsymbol{0}, \overbrace{\underbrace{\begin{bmatrix}\boldsymbol{W}_{t_0}(\boldsymbol{\theta}) & & \\ & \ddots & \\ & & W_{t_{T-1}}(\boldsymbol{\theta})\end{bmatrix}}_{\boldsymbol{W}(\boldsymbol{\theta})} + \sigma^2 \boldsymbol{I}_{nT}}^{\boldsymbol{\Sigma}(\boldsymbol{\theta}, \sigma^2)}\right)\,, \tag{19}$$

which is convenient for parameters inference.

Algorithm 2: Maximum Likelihood inference for the base model.

Input: $\boldsymbol{M}$, $\Delta \boldsymbol{y}$

Output: $\hat{\boldsymbol{\theta}}^p_{ML}$

$\hat{\boldsymbol{\theta}}^p_{ML} \underset{\boldsymbol{\theta}_k}{\leftarrow} \underset{\substack{\text{subject to}\\ \boldsymbol{\theta}\geq 0;\sigma^2\geq 0}}{\text{argmin}} \left\{log(|\boldsymbol{W}_*|) + (\Delta\boldsymbol{y} - \boldsymbol{M}\boldsymbol{\theta})'\boldsymbol{W}_*^{-1}(\Delta\boldsymbol{y} - \boldsymbol{M}\boldsymbol{\theta})\right\}$

1.4 Maximum Likelihood (ML)

We infer the parameters $(\boldsymbol{\theta}, \sigma^2)$ of Eq. (19) with a maximum likelihood approach, that is we solve the following constrained optimization problem

$$\hat{\boldsymbol{\theta}}_{ML} \leftarrow \underset{\substack{\text{subject to}\\ \boldsymbol{\theta}\geq \boldsymbol{0};\sigma^2\geq 0}}{\text{argmin}} f(\boldsymbol{\theta}, \sigma^2)\,, \tag{20}$$

where the objective function is

$$f(\boldsymbol{\theta}, \sigma^2) = log(|\boldsymbol{W}_*|) + (d\boldsymbol{y} - \boldsymbol{M}\boldsymbol{\theta})'\boldsymbol{W}_*^{-1}(d\boldsymbol{y} - \boldsymbol{M}\boldsymbol{\theta})\,, \tag{21}$$

and we compactly write the diffusion matrix $\boldsymbol{W}_* = \boldsymbol{W}(\boldsymbol{\theta}, \sigma^2)$ as a function of the free parameters. Using the rules of matrix calculus [3], the partial derivatives of f w.r.t. $\boldsymbol{\theta}$ and σ^2 can be written as

$$\begin{aligned}\nabla_{\boldsymbol{\theta}} f(\boldsymbol{\theta}, \sigma^2) &= \nabla_{\boldsymbol{\theta}} log(|\boldsymbol{W}_*|) + d\boldsymbol{y}'\nabla_{\boldsymbol{\theta}}\boldsymbol{W}_*^{-1}d\boldsymbol{y} + 2\boldsymbol{\theta}\boldsymbol{M}'\boldsymbol{W}_*^{-1}\boldsymbol{M} + \\ &- 2(\boldsymbol{M}'\boldsymbol{W}_*^{-1} + \boldsymbol{\theta}'\boldsymbol{M}'\nabla_{\boldsymbol{\theta}}\boldsymbol{W}_*^{-1})d\boldsymbol{y} + \boldsymbol{\theta}'\boldsymbol{M}'\nabla_{\boldsymbol{\theta}}\boldsymbol{W}_*^{-1}\boldsymbol{M}\boldsymbol{\theta}\,,\end{aligned} \tag{22}$$

$$\begin{aligned}\nabla_{\sigma^2} f(\boldsymbol{\theta}, \sigma^2) &= \nabla_{\sigma^2} log(|\boldsymbol{W}_*|) + d\boldsymbol{y}'\nabla_{\sigma^2}\boldsymbol{W}_*^{-1}d\boldsymbol{y} + \\ &- 2\boldsymbol{\theta}'\boldsymbol{M}'\nabla_{\sigma^2}\boldsymbol{W}_*^{-1}d\boldsymbol{y} + \boldsymbol{\theta}'\boldsymbol{M}'\nabla_{\sigma^2}\boldsymbol{W}_*^{-1}\boldsymbol{M}\boldsymbol{\theta} \\ & tr\left(\boldsymbol{W}_*^{-1}\right) - (d\boldsymbol{y} - \boldsymbol{M}\boldsymbol{\theta})'\boldsymbol{W}_*^{-1}\boldsymbol{W}_*^{-1}(d\boldsymbol{y} - \boldsymbol{M}\boldsymbol{\theta})\,,\end{aligned} \tag{23}$$

where

$$\begin{aligned}&\frac{\partial}{\partial\theta_j}\boldsymbol{W}_*^{-1} = -\boldsymbol{W}_*^{-1}\frac{\partial}{\partial\theta_j}\boldsymbol{W}_*\boldsymbol{W}_*^{-1}\,, \quad \frac{\partial}{\partial\theta_j}\boldsymbol{W}_* = \boldsymbol{W}((\ldots, \underset{j}{1}, \ldots), 0)\,, \\ &\frac{\partial}{\partial\sigma^2}\boldsymbol{W}_*^{-1} = -\boldsymbol{W}_*^{-1}\boldsymbol{W}_*^{-1}\,, \quad \frac{\partial}{\partial\theta_j} log|\boldsymbol{W}_*| = tr\left(\boldsymbol{W}_*^{-1}\frac{\partial}{\partial\theta_j}\boldsymbol{W}_*\right)\,, \\ &\frac{\partial}{\partial\sigma^2} log|\boldsymbol{W}_*| = tr\left(\boldsymbol{W}_*^{-1}\right)\,.\end{aligned} \tag{24}$$

Then, we solve the optimization problem (20) by using the objective function (21) and its gradients (22)-(23) inside the `L-BFGS-B` optimization algorithm from the `optim()` function of the `stats` R package. The inference procedure is summarised in Algorithm 2.

1.5 Random-effects stochastic reaction networks

From Eq. (19) it can be seen that all the molecules $y_1, \ldots, y_n$ share the same parameter vector $\boldsymbol{\theta}$. In some cases it may happen that the molecules being analysed are drawn from a hierarchy of J different populations having different properties. In this case it might be of interest to quantify the population-average $\boldsymbol{\theta}$ and the subject-specific effects $\boldsymbol{u}$ around the average $\boldsymbol{\theta}$ for the description of the subject-specific dynamics. Therefore, to quantify the contribution of each subject $j = 1, \ldots, J$ on the process's dynamics we extended the LLA formulation of Eq. (19) by introducing random effects $\boldsymbol{u}$ for the J

distinct subjects on the parameter vector $\boldsymbol{\theta}$, leading to the following mixed-effects [4] formulation

$$\Delta \boldsymbol{y} = \underbrace{\begin{bmatrix} \boldsymbol{M}_1 & & \mathbf{0} \\ & \ddots & \\ \mathbf{0} & & \boldsymbol{M}_J \end{bmatrix}}_{\mathbb{M} \in \mathbb{R}^{n \times Jp}} \boldsymbol{u} + \boldsymbol{\varepsilon}\,, \quad \boldsymbol{u} \sim \mathscr{N}_{Jp}\left(\underbrace{\mathbf{1}_J \otimes \boldsymbol{\theta}}_{\boldsymbol{\theta}_u}, \boldsymbol{I}_J \otimes \underbrace{\begin{bmatrix} \tau_1^2 & & \mathbf{0} \\ & \ddots & \\ \mathbf{0} & & \tau_p^2 \end{bmatrix}}_{\boldsymbol{\Delta}_u} \right), \qquad (25)$$
$$\boldsymbol{\varepsilon} \sim \mathscr{N}(\mathbf{0}, \boldsymbol{\Sigma}(\boldsymbol{\theta}, \sigma^2))\,,$$

where $\mathbb{M}$ is the block-diagonal design matrix for the random effects $\boldsymbol{u}$ centered in $\boldsymbol{\theta}$, and each block $\boldsymbol{M}_j$ is subject-specific. As in the case of the null model of Eq. (19), to explain additional noise of the data and to avoid singularity of the stochastic covariance matrix $\boldsymbol{W}(\boldsymbol{\theta})$ we added to its diagonal a small unknown quantity σ^2 which we infer from the data. In order to infer the maximum likelihood estimator $\hat{\boldsymbol{\psi}}$ for $\boldsymbol{\psi} = (\boldsymbol{\theta}, \sigma^2, \tau_1^2, \dots, \tau_p^2)$ we developed an efficient expectation-maximization E-M algorithm where $\Delta \boldsymbol{y}$ and $\boldsymbol{u}$ take the roles of the observed and latent states respectively. Under this framework

$$\begin{aligned} p(\boldsymbol{u}|\Delta\boldsymbol{y}) &\propto_{\boldsymbol{u}} p(\Delta\boldsymbol{y}|\boldsymbol{u})p(\boldsymbol{u}) \\ \propto_{\boldsymbol{u}} exp &\left(-\frac{1}{2}\boldsymbol{u}'(\mathbb{M}'\boldsymbol{\Sigma}^{-1}(\boldsymbol{\theta},\sigma^2)\mathbb{M} + \boldsymbol{\Delta}_u^{-1})\boldsymbol{u} + \boldsymbol{u}'(\mathbb{M}'\boldsymbol{\Sigma}^{-1}(\boldsymbol{\theta},\sigma^2)\Delta\boldsymbol{y} + \boldsymbol{\Delta}_u^{-1}\boldsymbol{\theta}_u) \right)\,, \end{aligned} \qquad (26)$$

and therefore

$$\boldsymbol{u}|\Delta\boldsymbol{y} \sim \mathscr{N}_{Jp}(E_{\boldsymbol{u}|\Delta\boldsymbol{y};\boldsymbol{\psi}}[\boldsymbol{u}], V_{\boldsymbol{u}|\Delta\boldsymbol{y};\boldsymbol{\psi}}(\boldsymbol{u}))\,, \qquad (27)$$

where

$$\begin{aligned} E_{\boldsymbol{u}|\Delta\boldsymbol{y};\boldsymbol{\psi}}[\boldsymbol{u}] &= V_{\boldsymbol{u}|\Delta\boldsymbol{y};\boldsymbol{\psi}}(\boldsymbol{u}) \left(\mathbb{M}'\boldsymbol{\Sigma}^{-1}(\boldsymbol{\theta},\sigma^2)\Delta\boldsymbol{y} + \boldsymbol{\Delta}_u^{-1}\boldsymbol{\theta}_u\right)\,, \\ V_{\boldsymbol{u}|\Delta\boldsymbol{y};\boldsymbol{\psi}}(\boldsymbol{u}) &= \left(\mathbb{M}'\boldsymbol{\Sigma}^{-1}(\boldsymbol{\theta},\sigma^2)\mathbb{M} + \boldsymbol{\Delta}_u^{-1}\right)^{-1}\,. \end{aligned} \qquad (28)$$

Also, the joint log-likelihood of $\Delta\boldsymbol{y}$ and $\boldsymbol{u}$ is given by

$$\begin{aligned} l(\Delta\boldsymbol{y},\boldsymbol{u};\boldsymbol{\psi}) &\propto_{\psi} l(\Delta\boldsymbol{y}|\boldsymbol{u};\boldsymbol{\psi}) + l(\boldsymbol{u};\boldsymbol{\psi}) \\ \propto_{\boldsymbol{\psi}} -\frac{1}{2}log|\boldsymbol{\Sigma}(\boldsymbol{\theta},\sigma^2)| &- \frac{1}{2}(\Delta\boldsymbol{y} - \mathbb{M}\boldsymbol{u})'\boldsymbol{\Sigma}^{-1}(\boldsymbol{\theta},\sigma^2)(\Delta\boldsymbol{y} - \mathbb{M}\boldsymbol{u}) + \\ -\frac{1}{2}log|\boldsymbol{\Delta}_u| &- \frac{1}{2}(\boldsymbol{u} - \boldsymbol{\theta}_u)'\boldsymbol{\Delta}_u^{-1}(\boldsymbol{u} - \boldsymbol{\theta}_u)\,, \end{aligned} \qquad (29)$$

which only depends on $\boldsymbol{u}$ linearly via its first two-order conditional moments of Eq. (28). Therefore, it follows for the E-step function that

$$\begin{aligned} Q(\boldsymbol{\psi}|\boldsymbol{\psi}^*) = E_{\boldsymbol{u}|\Delta\boldsymbol{y};\boldsymbol{\psi}^*}[l(\Delta\boldsymbol{y},\boldsymbol{u};\boldsymbol{\psi})] &= -\frac{1}{2}log|\boldsymbol{\Sigma}(\boldsymbol{\theta},\sigma^2)| \\ -\frac{1}{2}\{\Delta\boldsymbol{y}'\boldsymbol{\Sigma}^{-1}(\boldsymbol{\theta},\sigma^2)\Delta\boldsymbol{y} &- 2E_{\boldsymbol{u}|\Delta\boldsymbol{y};\boldsymbol{\psi}^*}[\boldsymbol{u}]'\mathbb{M}'\boldsymbol{\Sigma}^{-1}(\boldsymbol{\theta},\sigma^2)\Delta\boldsymbol{y} + \\ +\mathtt{tr}\left(\mathbb{M}'\boldsymbol{\Sigma}^{-1}(\boldsymbol{\theta},\sigma^2)\mathbb{M}[V_{\boldsymbol{u}|\Delta\boldsymbol{y};\boldsymbol{\psi}^*}(\boldsymbol{u})\right. &+ \left.E_{\boldsymbol{u}|\Delta\boldsymbol{y};\boldsymbol{\psi}^*}[\boldsymbol{u}]E_{\boldsymbol{u}|\Delta\boldsymbol{y};\boldsymbol{\psi}^*}[\boldsymbol{u}]']\right)\} + \\ -\frac{1}{2}log|\boldsymbol{\Delta}_u| - \frac{1}{2}\mathtt{tr}(\boldsymbol{\Delta}_u^{-1}[V_{\boldsymbol{u}|\Delta\boldsymbol{y};\boldsymbol{\psi}^*}(\boldsymbol{u}) &+ E_{\boldsymbol{u}|\Delta\boldsymbol{y};\boldsymbol{\psi}^*}[\boldsymbol{u}]E_{\boldsymbol{u}|\Delta\boldsymbol{y};\boldsymbol{\psi}^*}[\boldsymbol{u}]']) + \\ +E_{\boldsymbol{u}|\Delta\boldsymbol{y};\boldsymbol{\psi}^*}[\boldsymbol{u}]'\boldsymbol{\Delta}_u^{-1}\boldsymbol{\theta}_u &- \frac{1}{2}\boldsymbol{\theta}_u'\boldsymbol{\Delta}_u^{-1}\boldsymbol{\theta}_u\,. \end{aligned} \qquad (30)$$

The gradient of $Q(\boldsymbol{\psi}|\boldsymbol{\psi}^*)$ is defined by the following partial derivatives

$$\begin{aligned}
\frac{\partial}{\partial\theta_j}Q(\boldsymbol{\psi}|\boldsymbol{\psi}^*) &= -\frac{1}{2}\mathtt{tr}\left(\boldsymbol{\Sigma}^{-1}(\boldsymbol{\theta},\sigma^2)\frac{\partial}{\partial\theta_j}\boldsymbol{\Sigma}(\boldsymbol{\theta},\sigma^2)\right)+\\
&-\frac{1}{2}\Big\{-\Delta\boldsymbol{y}'\boldsymbol{\Sigma}^{-1}(\boldsymbol{\theta},\sigma^2)\frac{\partial}{\partial\theta_j}\boldsymbol{\Sigma}(\boldsymbol{\theta},\sigma^2)\boldsymbol{\Sigma}^{-1}(\boldsymbol{\theta},\sigma^2)\Delta\boldsymbol{y}+\\
&+2E_{\boldsymbol{u}|\Delta\boldsymbol{y};\boldsymbol{\psi}^*}[\boldsymbol{u}]'\mathbb{M}'\boldsymbol{\Sigma}^{-1}(\boldsymbol{\theta},\sigma^2)\frac{\partial}{\partial\theta_j}\boldsymbol{\Sigma}(\boldsymbol{\theta},\sigma^2)\boldsymbol{\Sigma}^{-1}(\boldsymbol{\theta},\sigma^2)\Delta\boldsymbol{y}+\\
&+\mathtt{tr}\left(-\mathbb{M}'\boldsymbol{\Sigma}^{-1}(\boldsymbol{\theta},\sigma^2)\frac{\partial}{\partial\theta_j}\boldsymbol{\Sigma}(\boldsymbol{\theta},\sigma^2)\boldsymbol{\Sigma}^{-1}(\boldsymbol{\theta},\sigma^2)\right)\mathbb{M}\Big[V_{\boldsymbol{u}|\Delta\boldsymbol{y};\boldsymbol{\psi}^*}(\boldsymbol{u})\\
&+E_{\boldsymbol{u}|\Delta\boldsymbol{y};\boldsymbol{\psi}^*}[\boldsymbol{u}]E_{\boldsymbol{u}|\Delta\boldsymbol{y};\boldsymbol{\psi}^*}[\boldsymbol{u}]'\Big]\Big\}+\\
&+E_{\boldsymbol{u}|\Delta\boldsymbol{y};\boldsymbol{\psi}^*}[\boldsymbol{u}]'\boldsymbol{\Delta}_u^{-1}\frac{\partial}{\partial\theta_j}\boldsymbol{\theta}_u-\boldsymbol{\theta}_u'\boldsymbol{\Delta}_u^{-1}\frac{\partial}{\partial\theta_j}\boldsymbol{\theta}_u\,,
\end{aligned} \tag{31}$$

$$\begin{aligned}
\frac{\partial}{\partial\tau_j}Q(\boldsymbol{\psi}|\boldsymbol{\psi}^*) &= -\frac{1}{2}\mathtt{tr}\left(\boldsymbol{\Delta}_u^{-1}\frac{\partial}{\partial\tau_j}\boldsymbol{\Delta}_u^{-1}\right)+\\
&-\frac{1}{2}\mathtt{tr}\left(\boldsymbol{\Delta}_u^{-1}\frac{\partial}{\partial\tau_j}\boldsymbol{\Delta}_u^{-1}\boldsymbol{\Delta}_u^{-1}\left[V_{\boldsymbol{u}|\Delta\boldsymbol{y};\boldsymbol{\psi}^*}(\boldsymbol{u})+E_{\boldsymbol{u}|\Delta\boldsymbol{y};\boldsymbol{\psi}^*}[\boldsymbol{u}]E_{\boldsymbol{u}|\Delta\boldsymbol{y};\boldsymbol{\psi}^*}[\boldsymbol{u}]'\right]\right)+\\
&-E_{\boldsymbol{u}|\Delta\boldsymbol{y};\boldsymbol{\psi}^*}[\boldsymbol{u}]'\boldsymbol{\Delta}_u^{-1}\frac{\partial}{\partial\tau_j}\boldsymbol{\Delta}_u^{-1}\boldsymbol{\Delta}_u^{-1}\theta_u+\frac{1}{2}\boldsymbol{\theta}_u'\boldsymbol{\Delta}_u^{-1}\frac{\partial}{\partial\tau_j}\boldsymbol{\Delta}_u^{-1}\boldsymbol{\Delta}_u^{-1}\boldsymbol{\theta}_u\,,
\end{aligned} \tag{32}$$

$$\begin{aligned}
\frac{\partial}{\partial\sigma^2}Q(\boldsymbol{\psi}|\boldsymbol{\psi}^*) &= -\frac{1}{2}\mathtt{tr}(\boldsymbol{\Sigma}^{-1}(\boldsymbol{\theta},\sigma^2))-\frac{1}{2}\Big\{-\Delta\boldsymbol{y}'\boldsymbol{\Sigma}^{-1}(\boldsymbol{\theta},\sigma^2)\boldsymbol{\Sigma}^{-1}(\boldsymbol{\theta},\sigma^2)\Delta\boldsymbol{y}+\\
&+2E_{\boldsymbol{u}|\Delta\boldsymbol{y};\boldsymbol{\psi}^*}[\boldsymbol{u}]'\mathbb{M}'\boldsymbol{\Sigma}^{-1}(\boldsymbol{\theta},\sigma^2)\boldsymbol{\Sigma}^{-1}(\boldsymbol{\theta},\sigma^2)\Delta\boldsymbol{y}+\\
&+\mathtt{tr}\left(-\mathbb{M}'\boldsymbol{\Sigma}^{-1}(\boldsymbol{\theta},\sigma^2)\boldsymbol{\Sigma}^{-1}(\boldsymbol{\theta},\sigma^2)\mathbb{M}\left[V_{\boldsymbol{u}|\Delta\boldsymbol{y};\boldsymbol{\psi}^*}(\boldsymbol{u})+E_{\boldsymbol{u}|\Delta\boldsymbol{y};\boldsymbol{\psi}^*}[\boldsymbol{u}]E_{\boldsymbol{u}|\Delta\boldsymbol{y};\boldsymbol{\psi}^*}[\boldsymbol{u}]'\right]\right)\Big\}\,.
\end{aligned} \tag{33}$$

In the E-M algorithm we iteratively update the E-function $Q(\boldsymbol{\psi}|\boldsymbol{\psi}^*)$ using the current estimate $\boldsymbol{\psi}^*$ of $\boldsymbol{\psi}$ and then we minimize the $-Q(\boldsymbol{\psi}|\boldsymbol{\psi}^*)$ w.r.t. $\boldsymbol{\psi}$. The E-M algorithm is run until a convergence criterion is met, that is when the relative errors of both the E-step function $Q(\boldsymbol{\psi}|\boldsymbol{\psi}^*)$ and the vector parameter $\boldsymbol{\psi}$ are lower than a predefined tolerance. Once we get the E-M estimate $\hat{\boldsymbol{\psi}}$ for the parameters we evaluate the goodness-of-fit of the mixed-model according to the conditional Akaike Information Criterion [5]. As every E-M algorithm, the choice of the starting point $\boldsymbol{\psi}_s$ is very important from a computational point of view. We chose as a starting point $\boldsymbol{\psi}_s = (\hat{\boldsymbol{\theta}}_{ML}, \hat{\sigma}^2_{ML}, \tau_1^2 = 0, \dots, \tau_p^2 = 0)$ where $(\hat{\boldsymbol{\theta}}_{ML}, \hat{\sigma}^2_{ML})$ is the optimum found in the fixed-effects LLA formulation of Eq. (19). This is a reasonable choice since we want to quantify how the dynamics $E_{\boldsymbol{u}|\Delta\boldsymbol{y};\hat{\boldsymbol{\psi}}}[\boldsymbol{u}]_j$ of each subject j departs from the average dynamics $\hat{\boldsymbol{\theta}}_{ML}$. The E-M pseudocode is given in Algorithm 3.

2 Data rescaling

2.1 Rhesus macaque study

Although the sample DNA amount was maintained constant during the whole experiment (200 ng for ZH33 and ZG66 or 500 ng for ZH17), the sample collected resulted in different magnitudes of total number of reads. Table 1 shows the total number of reads collected in each sample of the rhesus macaque clonal tracking dataset. This discrepancy makes

Algorithm 3: E-M inference algorithm for the mixed-effects model.

Input: $\boldsymbol{\psi}^* = (\hat{\boldsymbol{\theta}}_{ML}, \hat{\sigma}^2_{ML}, \tau_1^2 = 0, \ldots, \tau_p^2 = 0)$, $\boldsymbol{M}$, $\Delta \boldsymbol{y}$
Output: $\hat{\boldsymbol{\psi}}_{EM}$
chose a small tolerance `tol` and set $\epsilon = +\infty$;
while $\epsilon >$ `tol` **do**
  update $E_{\boldsymbol{u}|\Delta \boldsymbol{y};\boldsymbol{\psi}^*}[\boldsymbol{u}]$ and $V_{\boldsymbol{u}|\Delta \boldsymbol{y};\boldsymbol{\psi}^*}(\boldsymbol{u})$ as defined in Eq. (28) ;
  set to zero the negative elements of $E_{\boldsymbol{u}|\Delta \boldsymbol{y};\boldsymbol{\psi}^*}[\boldsymbol{u}]$;
  update $Q(\boldsymbol{\psi}|\boldsymbol{\psi}^*)$ and $\nabla_{\boldsymbol{\psi}^*} Q(\boldsymbol{\psi}|\boldsymbol{\psi}^*)$ according to Eqs. (31)-(33) ;
  set $\boldsymbol{\psi}_{old} \leftarrow \boldsymbol{\psi}^*$;
  update $\boldsymbol{\psi}^* \leftarrow \underset{\substack{\text{subject to} \\ \boldsymbol{\psi} \geq \boldsymbol{0}}}{\text{argmin}} \; -Q(\boldsymbol{\psi}|\boldsymbol{\psi}^*)$;
  update $\epsilon = |Q(\boldsymbol{\psi}_{old}|\boldsymbol{\psi}_{old}) - Q(\boldsymbol{\psi}^*|\boldsymbol{\psi}^*)|$;

$\hat{\boldsymbol{\psi}}_{EM} = \boldsymbol{\psi}^*$

		T	B	NK	M	G
ZH33	1	1465289	74735	135092	119331	2831
	2	225797	216844	335789	1035270	908685
	3	243986	413757	663184	886682	816990
	4.5	485542	479493	834064	985821	987171
	6.5	645005	676413	926089	895309	911637
	9.5	829073	962325	1057398	1229233	1220506
ZH17	1	51802	1347050	1288718	1351450	707382
	2	826190	1342700	1350703	1354355	1213749
	3	1303922	1347692	1338024	1347177	1283250
	4.5	190591	1206361	489098	572877	1195585
	6.5	887851	610999	1344488	381552	1339299
ZG66	1	752127	0	211350	13382	0
	2	692133	58890	308800	363310	145252
	3	339292	209137	424458	808404	704331
	4.5	617281	338977	718472	887183	897672

Table 1. Total number of reads (sum across the different clones) collected in each treated animal at each time point and for all the cell types.

all the samples not comparable across time and cell types. Therefore we rescaled the barcode counts according to

$$y_{ijk} \leftarrow y_{ijk} \cdot \frac{\min_{ij} \sum_c y_{ijc}}{\sum_c y_{ijc}}\,, \tag{34}$$

where y_{ijk} is the ijk-entry of the barcode matrix $\boldsymbol{Y}$ with dimensions (i, j, k) mapping respectively time, cell type and clone.

2.2 Genotoxicity study

Clonal tracking samples were collected under heterogeneous technical conditions that are reported in Table 2. The variability of these confounding factors makes clonal tracking samples not directly comparable across time and cell types. Here we consider the DNA amount (in nanograms), the vector copy number, the pool size and the PCR protocol (SLiM or Sonic-LAM) as potential confounders. By analogy to the SCS approach [6], we first evaluate and then remove the effect of the confounders from the observed data using

	PGK				LTR			
	DNA	VCN	PS	SD	DNA	VCN	PS	SD
Min.	8.64	1.31	1.000	60	8.64	0.240	1.000	189
1st Qu.	106.56	10.90	2.000	1969	94.50	5.320	1.000	1130
Median	200.00	13.59	2.000	5881	200.00	6.300	2.000	2973
Mean	181.07	12.80	1.964	9351	222.88	6.219	2.104	4695
3rd Qu.	200.25	13.90	2.000	14055	222.50	7.800	3.000	7390
Max.	973.00	27.00	3.000	49853	973.00	10.500	7.000	15375

Table 2. Mice study: Quartiles and range of the DNA amount, VCN, PS and SD for the $n = 242$ samples and separately for PGK (left) and LTR (right) treatments.

a regression approach. More precisely, we first perform a log-link Poisson regression on the collected cell counts $\boldsymbol{y}$ against the corresponding confounding factors, leading to the following model

$$log(\boldsymbol{\lambda}) = \boldsymbol{X}\boldsymbol{\beta}\,, \quad y_i \sim \texttt{Poisson}(\lambda_i)\,, \tag{35}$$

where y_i is the i-th component of $\boldsymbol{y}$, λ_i is the i-th component of $\boldsymbol{\lambda}$ for $i = 1, \ldots, n$, $\boldsymbol{X} = \begin{bmatrix}\mathbf{1} & \boldsymbol{X}_c\end{bmatrix}$ is the full design matrix including a term $\mathbf{1} \in \mathbb{R}^{n\times 1}$ for the intercept and a term $\boldsymbol{X}_c \in \mathbb{R}^{n\times 4}$ with confounder-specific columns. After having estimated the parameters $\hat{\boldsymbol{\beta}} = \left(\hat{\beta}_0, \hat{\boldsymbol{\beta}}_c'\right)'$ with a Fisher scoring algorithm, the rescaled clonal tracking data has been defined as the partial residuals corresponding to the confounders, that is

$$\boldsymbol{y}^{res} = \exp\left\{\log(\boldsymbol{y}) - \boldsymbol{X}_c\hat{\boldsymbol{\beta}}_c\right\}\,, \tag{36}$$

where $\hat{\boldsymbol{\beta}}_c$ are the optimal parameters for the confounders.

References

1. Sjöberg P, Lötstedt P, Elf J. Fokker–Planck approximation of the master equation in molecular biology. Computing and Visualization in Science. 2009;12(1):37–50.
2. Érdi P, Tóth J. Mathematical models of chemical reactions: theory and applications of deterministic and stochastic models. Manchester University Press; 1989.
3. Petersen KB, Pedersen MS. The Matrix Cookbook; 2012. Available from: `http://www2.compute.dtu.dk/pubdb/pubs/3274-full.html`.
4. Dobson AJ, Barnett AG. An Introduction to Generalized Linear Models. Chapman & Hall/CRC Texts in Statistical Science. CRC Press; 2018. Available from: `https://books.google.it/books?id=kIhnDwAAQBAJ`.
5. Vaida F, Blanchard S. Conditional Akaike Information for Mixed-Effects Models. Biometrika. 2005;92(2):351–370.
6. Del Core L, Cesana D, Gallina P, Secanechia YNS, Rudilosso L, Montini E, et al. Normalization of clonal diversity in gene therapy studies using shape constrained splines. Scientific Reports. 2022;12(1):3836.
